# Key components in the professional ethics of sign language interpreters in healthcare contexts: a qualitative study in Colombia

**DOI:** 10.1136/bmjopen-2024-086490

**Published:** 2025-02-11

**Authors:** Laura Catalina Izquierdo Martínez, Nora Ellen Groce, Samia Hurst, Angela Martínez-R, Jessica Cuculick, Minerva C Rivas Velarde

**Affiliations:** 1 Institute of Global Health, Faculty of Medicine, University of Geneva, Geneve, Switzerland; 2 School of Medicine and Health Sciences, Universidad del Rosario, Bogota, Colombia; 3 Leonard Cheshire Centre, Institute of Epidemiology and Healthcare, University College London, London, UK; 4 Institute for Ethics, History, and the Humanities, Faculty of Medicine, University of Geneva, Geneve, Switzerland; 5 NTID Department of Liberal Studies, Rochester Institute of Technology, Rochester, New York, USA; 6 Department of Radiology and Medical Informatics, Faculty of Medicine, University of Geneva, Geneve, Switzerland; 7 School of Health Science, University of Applied Sciences and Arts Western Switzerland, Geneva, Switzerland

**Keywords:** Decision Making, Disabled Persons, MEDICAL ETHICS, QUALITATIVE RESEARCH

## Abstract

**Abstract:**

**Objectives:**

To identified the core components of professional ethics for medical sign language interpreters and develop a framework based on empirical data from Colombian sign language (CSL) interpreters.

**Design:**

Purposive and snowball sampling methods were used in this qualitative study, which involved conducting semistructured interviews to CSL interpreters. Inductive data analysis was performed using the constant comparative method, where data collection and analysis occurred simultaneously. Transcriptional coding was performed line by line, and the data results were organised into themes and subthemes.

**Setting:**

The research was conducted in Colombia.

**Participants:**

A total of 17 CSL interpreters were included.

**Results:**

We identified key themes (confidentiality, privacy, professionalism, business practices and professional development). Our data analyses show the need for codes of conduct and establishment of professional codes of ethics for sign language interpreters working in a health context. The proposed framework addresses the challenges within the professional ethics of sign language interpreters in healthcare.

**Conclusions:**

These findings offer unique insights into the ethical experiences of CSL interpreters. This framework can be a valuable reference for interpreters facing ethical dilemmas. The clarity of ethical considerations is crucial for overcoming barriers to healthcare for the D/deaf population. The identified ethical issues underscore the necessity of education, training and the establishment of codes of ethics and legislation for sign language interpreters. These findings can serve as a foundational reference for crafting ethical guidelines for sign language interpreters in other low- and middle-income countries.

**STRENGTHS AND LIMITATIONS OF THIS STUDY**

- Perspectives and experiences of medical sign language interpreters in Colombia regarding the core components of professional ethics helped in generating rich data serving as a foundational reference for developing ethical guidelines.
- One-to-one semistructured interviews allowed deeper insight into participants’ experiences and perceptions.
- Inductive analysis allowed for a thorough investigation, offering a deeper and more comprehensive understanding of the research topic.
- The exclusive use of interviews limits the study to an initial understanding, emphasising the need for future research employing diverse methods for a more comprehensive perspective.
- The study is limited to the Colombian context, which may have unique cultural, legal and professional dynamics.

## Introduction

 Sign language interpretation functions to establish and optimise communication between D/deaf and hearing persons, acting as a bridge between both languages and cultures. The concept of professional ethics has particular importance in the sign language interpreters’ role in the health context;^1^ D/deaf persons are at risk of missing essential health information if interpretation is not done effectively and efficiently which can compromise the quality of care. Professional ethical standards ensure effective communication and protect the rights and well-being of the D/deaf individuals.

Deaf with capital ‘D’ is used when the person identifies himself/herself as a member of the deaf community and his/her language is sign language. The term deaf with lowercase ‘d’ is used when the person does not identify as culturally Deaf and has severe to profound hearing loss and uses spoken language, and ‘D/d’ is used to refer to both populations without any specific differentiation.^2^

The literature shows that D/deaf persons from low- and middle-income countries (LMICs) face significant health disparities compared with those living in high-income countries.^3^ Being D/deaf is a powerful predictor of social deprivation.^4^ Vulnerability factors, such as lack of social support, predispose people to the development of diseases, leading to negative effects on their health.^5^

The availability of interpreters and technological tools, like video remote interpreting (VRI), is an important determinant of health that influences the health outcomes of the D/deaf population.^6^ Access to sign language in healthcare settings is a fundamental right of D/deaf people and is embedded on the United Nations Convention on the Rights of Persons with Disabilities (UN CRPD) Article 25 ‘health’, which clearly states that persons with disabilities have the right to the enjoyment of the highest attainable standard of health without discrimination on the basis of disability.^7^

Sign language interpreters facilitate communication between D/deaf people and the hearing community. Their impact on the health outcomes of D/deaf people is significant, as they improve access to healthcare information, enhance communication, enable timely and accurate diagnoses and contribute to other key aspects of healthcare delivery.^8^ However, access to healthcare services and information for D/deaf people is frequently problematic.^9^,^14^ Because of the linguistic and cultural differences between D/deaf patients and non-deaf providers, trained, qualified and credentialled interpreters are of paramount importance to ensure quality and equity in communication.^15 16^ Often a D/deaf person has limited access to communication in healthcare settings because providers do not provide credentialled healthcare interpreters, largely because of a shortage of interpreters nationwide.^17^ This communication barrier decreases the ability of D/deaf people to understand and participate in decision-making about their healthcare and increases the likelihood that they will delay needed healthcare in the future.^15^

Yet in many LMICs, such as those in Latin America, difficulties exist in providing adequate certification for sign language interpreters. This is particularly true where education to train or certify sign language interpreters is not mandatory. It is estimated that in Mexico, for example, there are only 42 certified sign language interpreters in the entire country.^18^ In Paraguay, there are 37 certified interpreters.^19^ A comparable lack of professional interpreters is seen in Peru, Chile and Colombia.^20 21^

Certification for interpreters working with D/deaf individuals in medical settings should be limited to those who have completed a certain number of hours in sign language courses and who are well versed not only in medical terminology and signs but also in ethics and knowledge of the D/deaf community. This standard ensures that healthcare institutions and medical practitioners hire interpreters who are fully qualified to provide effective and culturally competent interpreting services. In many countries, medical interpretation is not available. There are no official degrees in interpretation endorsed or recognition from governmental or educational institutions, nor are there any official course of studies or qualifying exams.^22^ This situation is not unique to LMICs. Higher-income countries such as Spain and Canada also face shortage problems with the training and recruitment of enough certified interpreters.^23 24^

Other countries such as the USA and the UK have better regulations for sign language interpreters’ certification. In both, certification levels are based on skill sets and the number of years of experience. D/deaf people and healthcare institutions are encouraged to contract interpreting services which meet such established quality standards.^25^ Comparable training certification programmes are found in other countries as well. For example, in South Africa, interpreter training is currently offered by several university programmes, where interpreters are assessed based on language skills, content/message, interpreting technique and professional conduct and are accredited on a national basis.^26^

The National Association for the Deaf-Registry (NAD-RID) based in the USA has created the most well-known international code of professional standards of conduct. This code outlines the duties and responsibilities for interpreters in a range of contexts. It also promotes seven guiding principles of interpreter conduct: confidentiality, professionalism, conduct, respect for consumers, respect for colleagues, business practices and professional development.^27^ The NAD-RID guidelines are based on the codes of ethics for interpretation in healthcare from professional associations of spoken language interpreters, such as the National Council on Interpreting in Healthcare,^28^ the International Medical Interpreters Association^29^ and the California Healthcare Interpreters Association.^30^ These codes of conduct and standards of practice for medical interpreters’ work are designed to apply to interpretation for spoken languages and handle similar principles such as confidentiality, impartiality, respect, accuracy of the information, professionalism and advocacy when the patient’s health, well-being or dignity is at risk. In the absence of a code of ethics for medical sign language interpreters in a number of countries, including Colombia, interpreters are advised to adhere to international codes of ethics. Specifically, medical sign language interpreters in healthcare should reference and align with those established for spoken language interpreters.

Although there is an international framework in place that promotes a coherent and comprehensive set of professional standards of medical interpretation for sign language interpreters (NAD-RID code), compliance remains limited among healthcare institutions, policymakers, associations representing the D/deaf community and interpreters. Even countries without their own codes are not compliant with NAD-RID code. Contributing factors include inadequate training programmes, limited awareness of these standards and the lack of formal certification programmes in many regions. Weak enforcement mechanisms further hinder consistency, and in the absence of regulatory bodies, compliance relies on voluntary commitment rather than on enforceable requirements. Establishing criteria to allow D/deaf clients and healthcare workers and institutions to evaluate interpretation services is currently a critical missing component.

The absence of a Colombian public policy for sign language interpretation has a direct impact on the quality of the services provided. That is why this paper identified professional ethics core components of medical sign language interpreters in Colombia. We argue here that clarity on ethics will contribute to overcoming barriers to health for the D/deaf population. This paper provides a framework based on empirical data related to Colombian sign language (CSL) interpreters, designed to enhance ethical decision-making among interpreters that navigate into complex situations, encourage best practices and facilitate more culturally competent care. Building on existing international frameworks, this Colombia-specific addresses the cultural and legislative contexts essential for meeting the unique needs, values and preferences of the local D/deaf community. Additionally, it considers the variations in language, ensuring that interpretations are accurate and culturally appropriate. Colombia’s national sign language is a distinct language with its own structure and vocabulary, but it also includes regional variations, with unique dialects and local signs that vary across different parts of the country. This means that sign language interpreters must not only be proficient in the national language but also understand local dialects and signs, adapting their interpretation based on the person they are interpreting for. We believe this framework from Colombia may also be relevant to other LMICs seeking to establish a framework for helping interpreters make ethical decisions in real-world scenarios.

### Background

#### Sign language interpreters’ formation

Professional ethical standards for sign language interpreters refer to a set of guidelines and principles that govern the conduct and responsibilities of interpreters in their professional practice.^31^ In Colombia, there is no institutional or governmental policy that promotes comprehensive training and qualification in sign language interpreting.^32^ Indeed, until 2021, there was no formal training programme or accreditation system for interpreters. Today, professional-level training for sign language interpretation is offered by four universities in Colombia, but the first cohort of trained interpreters has not yet graduated. It remains uncertain if there are going to be formal exams or systems of assessing and evaluating competency and formally awarded accreditation for these programmes and certification for those who attend them.

Currently, most interpreters are trained by different Deaf person associations or local or national non-governmental organisations. Despite the creation of new established professional training programmes, most of which are privately offered, these programmes operate in parallel. These interpreters are not obligated to provide evidence of training or certification through exams to validate their competencies. Certificates issued by non-governmental organizations or Deaf associations simply indicate a level of sign language proficiency. At present, there are no clear guidelines or regulations defining what agencies or groups should do to guarantee the quality of interpreters’ education and their skill level when providing interpreting services.

Not following professional standards in sign language interpretation can lead to several serious issues, especially in healthcare. These include misunderstandings about diagnoses, treatments and medication instructions, which can result in patient harm; diminished trust due to breaches in confidentiality or failure to follow privacy protocols; and inconsistent service quality that varies between providers and settings.^3^

In Colombia, there is no sign language interpreters’ database of professional or practicing sign language interpreters who can, or routinely do, work in healthcare settings. Indeed, no database for general, non-medical interpreters exists either. In a healthcare context, in theory, interpreters must be familiar with medical terms, common medications, medical procedures and commonly asked questions. They should also possess knowledge of specialised signs and regional dialectal variations to ensure accurate interpretation and effective communication with D/deaf patients.^33 34^ The responsibility for determining competence to provide accurate medical interpretation and to accept a job rests with the interpreters themselves. Many sign language interpreters, while fluent in CSL, have not been specifically trained to work in specialised areas, such as healthcare, and lack knowledge of clinical care medical terminology or significant ethical concerns in a medical setting.

A lack of awareness of these issues or interpreters’ insufficient breadth of lexicon can have serious consequences for the health and well-being of persons with disabilities. This can lead not only to a D/deaf person lacking full understanding of medical findings and discussions about their health and this results in longer appointment times. If the interpretation is not going well, a visit may also be cut short or run out of time for the patient, and the D/deaf patient will be provided less information or support than they may need.

There are additional issues. Currently, many people in Colombia who work as interpreters are children of deaf adults and relatives who have grown up in Deaf households. As is true for D/deaf communities in many countries, in Colombia, this means that interpreters are well known within the D/deaf community and the need for rigorous professional standards in place to protect confidentiality is of particular importance.

Another issue is that many interpreters are members of the Jehovah’s Witness religion. This religious group is one of several evangelical faiths that have concentrated on teaching sign language to their hearing members,^34^ as part of an effort to gain converts from among the Deaf community. This initiative has resulted in an increase in the number of Jehovah’s Witnesses who know sign language and, thus, who now serve as interpreters. This focus on the D/deaf community reflects a religious dedication to altruistically assist the most needy, vulnerable or unfortunate people and reflects the assumption that D/deaf people automatically fall into this category.^34^ This presents challenges and, as will be discussed later in this paper, has raised a series of concerns within the D/deaf community itself.

#### Types of sign language interpretation

In Colombia, sign language interpretation is available either in-person, through VRI or a combination of both. In most cases, in-person interpreting services require D/deaf individuals to cover the costs themselves, unlike in some high-income countries, such as the USA, where funding options are more accessible. In contrast, VRI is intended to be a free service, funded by the Ministry of Information Technology and Communications. VRI uses a video camera on an electronic device to connect D/deaf and hearing individuals with a sign language interpreter via video call. Through this system, D/deaf individuals can request interpretation services for social, academic, medical or judicial purposes. However, while the VRI service itself is free, D/deaf individuals must use their own phones and internet to access it, potentially incurring personal costs for data or phone usage. This creates indirect expenses, making the service not entirely cost-free. So, while the service is free in terms of the actual communication, there are potential indirect costs that make it not entirely without expense.

For D/deaf individuals, the VRI has provided great opportunities since they help them receive healthcare through a competent interpreter. However, there is a digital divide faced by the D/deaf population when accessing VRI services. It has been observed that D/deaf people in rural areas have greater difficulty accessing the VRI due to connection difficulties. Even when the D/deaf individual has access to VRI, they still face different barriers in access to healthcare because they have to use their own devices or pay for their own internet packages. This leads not only to additional expenses but also to limitations and failures in connectivity that prevent adequate visibility of the interpreter and, therefore, to communication barriers between health personnel and the D/deaf person.

#### Hiring characteristics in the healthcare context

There are different ways to hire interpreters in the health sector. Some interpreters are hired by D/deaf clients, while others are hired and work in the VRI offices awaiting calls to provide their services. A few are hired and work on call for a hospital or clinic that elects to provide interpreting services in their facility. Some interpreters may work for all of these entities. Interpreters also provide their services to friends or relatives as a favour, without being paid.

Effective management of conflicts of interest is crucial for maintaining integrity, trust and transparency in the delivery of interpreting services. For this reason, many interpreters working in healthcare settings choose not to interpret for their own family members. However, maintaining this boundary can be challenging for interpreters, as the D/deaf community constitutes a socio-linguistic minority, often resulting in close relationships between its members and interpreters. It is also essential for interpreters to be aware that being D/deaf does not imply vulnerability and that they are not proxies for D/deaf people, despite having moral and professional responsibilities towards them.^35^ An additional ethical dilemma for interpreters is that they must balance getting paid for their services with simply offering assistance as a favour, which makes it difficult to establish professional boundaries.

Not maintaining professional boundaries can result in several unethical practices in interpretation, such as loss of objectivity, where personal feelings or relationships interfere with impartiality and affect the accuracy of the interpretation. Other issues include breaches of confidentiality and inappropriate involvement. For instance, if an interpreter shares details about a patient’s medical condition or treatment with someone not involved in the case, it could harm the patient and violate confidentiality.

There is variability in the skillset of interpreters because of the inadequate training and regulation. The lack of adequate professional training and solid regulation in hiring means that some interpreters provide much better services than others. For example, interpreters with comprehensive training in medical terminology and ethical practices may deliver accurate and culturally competent interpretations, ensuring effective communication in healthcare settings. In contrast, those without such training may struggle to convey critical information, leading to misunderstandings in diagnosis or treatment plans. Good or bad, institutions are often forced to hire sign language interpreters even if they are not considered competent by D/deaf clients or healthcare professions. This is compounded by the fact that there are not enough trained interpreters to provide services.

## Methods

### Positionality statement

The research team offered diverse perspectives on sign language interpretation in healthcare, with members from Colombia, Switzerland, England and the USA, specialising in global health, disability studies, Deaf studies, ethics and healthcare access. All researchers have experience working with vulnerable populations, and several of them have collaborated closely with sign language interpreters, Deaf communities, and local experts to ensure cultural and contextual sensitivity in the work.

Through a qualitative exploration with semistructured interviews, we identified professional ethics core components of medical sign language interpreters. Based on this information, we proposed an ethics framework to guide decision-making.

### Patient and public involvement

This research is part of a more extensive study titled ‘Assessing the Impact of Video Remote Sign Language Interpreting in Healthcare: Linking Disability Studies with Empirical Challenges of Public Health Research’ (https://data.snf.ch/grants/grant/186035). The research questions of this study were as follows: How do D/deaf persons experience access to healthcare? Where and how does exclusion occur? How an intervention based on information and communication technology responds to the structural and societal factors that in conjuction with impairments determine how an individual experiences health and disability? This large international research project applies participatory research from the design to the implementation and is overseen by a steering committee, which includes disabled persons’ organisations such as World Federation of the Deaf, Instituto Nacional de Sordos, Federación Nacional de Sordos de Colombia, D/deaf scientists working on health-related issues, leading global health disability scholars, and leaders of the association of Colombian sign language interpreters. This steering committee held annual meetings to guide the direction of the study providing invaluable insights into and critical feedback on project outcomes, within the broader matrix of the Deaf community, disability studies and global health.

### Recruitment and sample

In Colombia, there is no database of professional or practicing sign language interpreters who work in healthcare settings. Therefore, general context interpreters were invited to participate through various channels, including email outreach, word-of-mouth within Deaf and interpreter associations nationwide, and information dissemination via the National Federation of the Deaf in Colombia and the relay institution centre (‘*Centro de relevo*’). Permission was obtained from the relay institution to access a list of potential participants, which included contact information such as names and email addresses. Of the 21 individuals contacted, 17 agreed to participate.

Interested individuals who reached out to research team were sent a study invitation and a participant information leaflet via email. Snowball sampling was employed as recruited participants were asked to forward study details (including background, aims and objectives) to those who may be eligible, for example, colleagues. Purposive sampling was used in the later stages of recruitment to capture variation that could influence perspectives, for example, interpreters who work in-person and via VRI were selected to provide maximum variation sampling across the types of medical interpretation and geographical location.

### Data collection and processing

Semistructured interviews were conducted remotely in Spanish by a female trained research assistant (LCIM) using video conferencing software (Zoom, San Jose, California). Data collection took place between August 2022 and February 2023 and interviews lasted between 30 and 80 min. An interview guide was developed based on the literature, researchers’ experiences and informal conversations with CSL interpreters (table 1).

**Table 1:** Interview guide

Main topic	Questions
Career pathway	What is your experience as an interpreter?
	Where did you train as an interpreter?
	What motivated you to learn CSL?
Health context experience	How do you feel when attending the interpretation of a medical appointment?
	Did you have any training or preparation process to assist in the interpretation of medical appointments?
	How do you observe the physician-deaf person interaction during the consultation?
	How do you judge that the interpretation in CSL is well done?
	How do you make sure that the person understood all the information?
	Have you observed any differences between in-person and VRI interpretation?

CSL, Colombian sign language; VRI, video remote interpreting

Interviews were recorded and transcribed. Video recording was crucial because many CSL interpreters used signing to convey their thoughts and emotions, and these signs were codified in the transcription. An initial translation of the quotations from Spanish to English was conducted by MCRV, who is proficient in both languages. Following this, SH, JC and NEG reviewed the quotations and identified any unclear sections for further review. Field notes were also made during the interviews. Data collected from individual sign language interpreters included their educational background in sign language, specifically the institution or organisation through which they received their training, the number of years of experience as interpreters and their current workplace. Topics included why individuals were motivated to work as sign language interpreters, training processes, ethical considerations in their professional practice, and experience in interpreting in a healthcare context. Interviews took place until data saturation was achieved. Data saturation was determined when the research team considered the data sufficiently robust and suspended the recruitment. Analysis checked the redundancy of the emergent themes.

### Analysis

Data analysis was performed using the constant comparative method, where data collection and analysis occurred simultaneously.^36^ In the data analysis process, the researcher (LCIM) first read the transcripts several times to become familiar with the content. Recordings were transcribed verbatim, and field notes were converted into data documents using NVivo Data Analysis Software V.14 (NVivo, Melbourne, AU). Subsequently, the two researchers developed a codebook (LCIM, MCRV; authors of this manuscript). All personal and institutional identifying data were removed from all interview transcripts before coding and analysis. Transcriptional coding was performed line by line, and the data results were organised into themes and subthemes. One participant required a second interview because the team felt that certain topics related to ethics in interpretation needed to be explored in greater depth, resulting in a total of 18 interviews. Inductive analysis was also performed.

After every three to four interviews, the researcher team held review sessions to examine the interpretations and discuss any discrepancies during coding and analysis of the results (LCIM, MCRV, AM-R). As the interviews were coded, the codebook was expanded to ensure that novel responses were captured for the analysis. We assigned participants a pseudonym, which we use when attributing quotes. Investigator triangulation during data analysis further addressed credibility and confirmability; it also ensured interpretations were not based on the first author’s understandings alone. To improve the intercoder reliability, emerging themes were collectively discussed with the full research team. Additionally, we triangulated the information with another qualified interpreter to ensure that the meaning was accurately captured and consistent across interpretations.

Based on the results of the interviews, we aligned our findings with established international guidelines and practices, including the NAD-RID code, National Council on Interpreting in Healthcare, and International Medical Interpreters Association. Consolidated criteria for Reporting Qualitative Research Guidelines was also used to ensure a rigorous and transparent reporting process for qualitative research. This framework also helped organise and report important aspects of the research team, study methods, context of the study, findings, analysis and interpretations.^37^

## Results

The final sample was composed of 17 participants. Slightly more than half were males (n=9) and the rest were females (n=8), and the majority of the participants (n=15) had more than 6 years’ experience as sign language interpreters. Approximately half of the participants worked using VRI (n=9). The sample included eight interpreters who work in Bogotá, while the remaining number worked in different cities of Colombia (table 2).

**Table 2:** Participants’ characteristics (n=17)

	Type of sign language interpretation		
	VRI (n=9)	In-person (n=8)	Total (n=17)
Gender			
Male	3	6	9
Female	6	2	8
Years of experience			
0–5	1	1	2
6–10	4	2	6
11–20	4	2	6
>20	0	3	3
Place of work			
Bogotá	3	5	8
Antioquia	1	3	4
Cesar	1	0	1
Cauca	1	0	1
Sucre	1	0	1
Atlántico	1	0	1
Santander	1	0	1

VRIvideo remote interpreting

**Figure 1:**
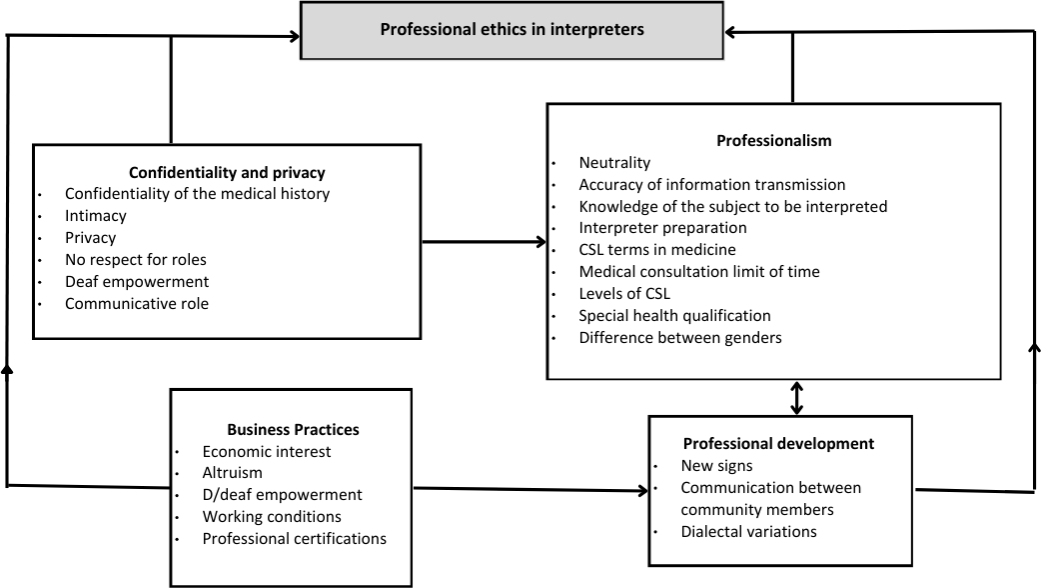
Themes and subthemes of professional ethics as perceived by sign language interpreters in our study. CSL, Colombian sign language.

The analysis yielded four themes that show key challenges and core components of professional ethics as perceived by sign language interpreters in a healthcare context (figure 1).

### Confidentiality and privacy

#### Confidentiality of the medical history

Because of the personal nature of the healthcare encounter, confidentiality and patient privacy was one of the principal themes. It is an ethical principle that puts interpreters under obligation not to disclose to anyone information that has been learnt during the performance of their duties as an interpreter.

Although most of the sign language interpreters do not have any medical training, the idea of confidentiality was an important subject during their interpreter training, even if it was not specifically medically focused. Interpreters reported that D/deaf patients prefer to call a trusted interpreter when attending medical appointments. Interpreters face the D/deaf person’s distrust due to the fear that the information revealed in the medical appointment (especially in mental health appointments) will be disclosed.

For example, one interpreter reported that:

…and she said [the Deaf patient]: “I don’t want to go with you; I mean, I don’t have anything against you, it’s not that I have something against you, but I don’t have the trust for you to know, and I’m afraid that later they [the Deaf community] will be talking about it”. (Female 16)

Generally, interpreters do not know the D/deaf person when communicating via VRI. A minority mentioned that not knowing the interpreter and the distance imposed by the use of VRI makes medical consultations feel more confidential because a D/deaf person is then more confident that the information he or she provides will not be disclosed.

In fact, it must be more comfortable for the D/deaf person because maybe I don’t know him, he doesn’t know me. It’s fine. At least it can be more fluid. (Female 16)

#### Intimacy

Another area of concern in many medical visits reported by interpreters begins the minute patients are asked by clinicians to undress. When a D/deaf person is asked to undress, the interpreters interviewed mentioned they feel uncomfortable due to the lack of academic training and experience. They mentioned that the feeling of discomfort increases when the D/deaf person is a close family member or friend.

…when the doctor told her [the D/deaf patient] she had to undress and put on a gown, the woman turned red with embarrassment. And she said to me, “What a shame that you are going to see me naked.” (Male 08)

#### Privacy

In these situations, the majority of the interpreters interviewed mentioned that they face a dilemma between deciding if they should stay and be communication facilitators or respect the D/deaf person’s privacy and withdraw during the examination. This means that there is no established set of professional guidelines to help guide the D/deaf person’s decision and preferences and the interpreter’s actions during a medical consultation. Only two of the interpreters mentioned that they make prior agreements with the D/deaf person, asking if they want him/her to be present or not during a physical examination.

…when a doctor tells you, “Take off your clothes,” generally they [the D/deaf patients] take off all of their clothes. I said, “what do I do? Do I get out? Do I stay? I have to be here. If I get out, how will the doctor interact with the patient?” (Male 14)

Also, most interpreters expressed discomfort when observing that the D/deaf looked embarrassed or ashamed because a non-clinician is seeing them naked.

…but it was very awkward because he [the D/deaf patient] had to undress in front of me, let’s say that was the one that had the biggest impact on me because wow! He was in something very intimate, that is, not only the space of his nudity, also the deep topic. (Female 01)

When confronted with these situations, VRI interpreters showed relief at not being physically present and thus being able to ensure the privacy of the D/deaf person, as the camera can be turned in another direction or simply turned it off.

10 interpreters surveyed mentioned that their primary motivation for learning sign language was driven by religious beliefs (eg, Jehovah’s Witnesses), with a strong desire to assist those in need. They reported that training as an interpreter based on religion caused them to have conflicts when interpreting in medical consultations where intimate information of the D/deaf person is discussed, such as cytology or urology appointments. Religious beliefs may conflict with medical practices or procedures, such as those involving reproductive health, end-of-life decisions or blood transfusions. This can create internal conflicts if interpreters feel their beliefs clash with the medical information they are asked to convey.

#### No respect for roles

In addition, interpreters highlighted the importance of professional limits in healthcare settings. One interpreter stated that he avoids accepting assignments involving people he knows personally as it becomes challenging to maintain professional boundaries and confidentiality of the medical history. Others mentioned that they often find themselves interpreting in healthcare settings for friends or family members.

Some of those interviewed expressed the desire to accompany D/deaf individuals to medical appointments, viewing it as an act of kindness, even without monetary compensation. However, some interpreters reported that this sometimes leads to unexpected calls from D/deaf friends or family members while at the doctor’s office. Such instances create tension for interpreters, torn between their connection to the D/deaf community and the need to maintain a professional and emotional distance.

Another day it happened to me that I was very quiet at home and a D/deaf girl I knew called me by video call, she was from [mentions her place of work] […] that is, on a Saturday 10 in the morning, she called me and I answered her. She [the D/deaf patient] was in the office with the doctor, and me [surprised face]. The doctor asking her. It was a routine appointment for a cytology and I was not informed previously. (Female 16)

### Business practices

Interpreters are expected to conduct their business professionally. They are entitled to a living wage based on their qualifications and expertise. Interpreters are also entitled to working conditions conducive to effective provision of services.

#### Economic interest

The sign language interpreters emphasised the necessity of accepting job offers that align with their skills, experience, knowledge and contextual abilities to ensure optimal performance in their work, despite this, many still accept positions that exceed their ability for which to provide accurate translations. As a result, interpreters often find themselves compelled to accept any job offered, competing for the best paying jobs even if they do not have adequate interpreting skills for that specific situation. Several factors contribute to the precariousness of hiring interpreters, including poor labour legislation and regulations, and lack of education and training in interpretation in specific contexts.

So, if they are going to pay me for one hour of service $col 250.000 [US$ 50.00], we line up. So, if there are two and there are three, how much am I going to earn? But how much of what is happening there in the communication, I am competent and I am doing it with an ethical responsibility as well? How much of that? […] when I accepted an interpreting service for a certain context, what competencies do I possess? What experience do I have to say yes or no? (Male 10)

#### Altruism

The interpreters justified the fact that they were entitled to secure equitable compensation for the interpreting services they deliver. However, the lines are also blurred regarding payment for such interpreting services. It is the responsibility of the D/deaf individuals to cover the costs of in-person interpreting. Interpreters may choose not to charge for their services, or hesitate to charge even if they need the money. Interpreters may face difficulties when trying to charge a D/deaf person with whom they are close for the service, as the close D/deaf individual might feel upset about it. For interpreters, this becomes a moral dilemma whether to offer an altruistic service or to charge for their knowledge and skills, especially if the person with whom they are close to has limited financial resources

Well, time also means money. I don’t know if other interpreters even charge for breathing […] and sometimes you can, but not always, and when you can’t, it bothers them [the Deaf community]. (Female 16)

Interpreters see themselves as advocates, empowering the D/deaf person to assert their rights while providing interpreting services. This empowerment is a key role. If they fulfil their role as interpreters, they empower the D/deaf person. Communication in the healthcare setting poses challenges to this advocacy and empowerment. Healthcare professionals often speak directly to the interpreter rather than directly addressing the D/deaf individual. The interpreters reported that they strive to uphold their role of empowerment and bridge the communication gap between healthcare providers and the D/deaf community.

All the time the doctors were not addressing the patient, but me. As if I were a relative of the patient. So many times, they gave me explanations and even though I interpreted them simultaneously. No, there is no individualized attention because the D/deaf patient realizes that the doctor is addressing the other person and not him. (Male 13)

#### Working conditions

Frequently, agreements for payment are made verbally without any formal documentation. Due to the lack of legal regulations on the working conditions for sign language interpreters, these verbal agreements may be unenforceable. One interpreter highlighted some of the challenges faced when trying to collect payment.

The interpreting service I provided during the COVID period—they didn’t pay me for that. They told me, “Come, work.” I went there, and at the end of the day, they didn’t pay me. So many times, as an interpreter, you are also exposed […] They took advantage of me and used me when situations like that came up. (Male 07)

Providing support to colleagues is a crucial aspect of an interpreter’s role. VRI interpreters mentioned that accuracy of the signs used is a professional concern. A positive aspect highlighted by interpreters at the relay institution centre is that colleagues support one another when they have doubts about signs, often engaging in a consultation process. Additionally, a quality assurance team composed of both D/deaf and hearing persons with interpreting experience periodically verifies and evaluates the accuracy and appropriateness of the information conveyed in sign language.

The support of the relay centre […] I have the support of my bosses, the support of the coordinators, of a group of great colleagues who give me signs feedback. I have the support of a quality group. (Female 04)

#### Professional certifications

All interpreters report that entities, institutions or D/deaf individuals often hire anyone with some levels of sign language as an interpreter due to the varied quality and diversity of existing training programmes. However, this does not necessarily guarantee that these individuals possess an acceptable level of competency.

The certificates issued by the National Federation of the Deaf, has a stamp that says: “no, this does not accredit you as an interpreter” but the same public entities or state entities, the entities at the departmental and municipal level in each region hire you with that modality. You are going to be hired as an interpreter. But they are hiring me with documents that say I am not an interpreter. A totally contradictory thing, right? (Male 10)

### Professional development

Interpreters are required to cultivate and sustain their interpreting proficiency by continuously expanding their knowledge and honing their skills. This expectation comes from the D/deaf community, which often evaluates an interpreter’s effectiveness based on their point of view and anecdotal observations. Interpreters highlighted the importance of staying consistently informed about emerging new signs, as they regularly interact with the D/deaf community and keep up with updates on social media.

For example, when COVID happened, COVID did not exist and therefore did not have a sign. So, within the community this thing came up and we had to sign it, the D/deaf community signs it and they are responsible of making these publications. (Female 06)

Most of the interviewees highlighted the importance of reaching agreements with the D/deaf person before providing their services. These agreements entail defining the signs to be used for specific words, depending on the city, institution or context. In some cases, these agreements are made in collaboration with sign language interpreters or D/deaf linguistic experts. However, more commonly, interpreters directly negotiate the agreements with the D/deaf patients.

Interpreters also report challenges due to the lack of standardisation for newly created signs. The absence of up-to-date bibliographic materials creates difficulties with the dialectal variations of signs between the different geographic regions of the country which makes it difficult for the same interpreters to work in the different regions of Colombia.

For example, in Bogotá a sign is created, in Medellín [3] they are thinking, let’s do it like this. In Cali how is it being thought? we don’t know, but in Medellín it is being done like this. There is no standardization, which is something that has also been lacking in the country. (Male 10)

Interpreters are often part of informal groups. For example, there are online groups or informal in-person meetings in which they engage in discussions on diverse topics. These groups include D/deaf people, sign language interpreters and some linguists. When interpreters find ethical dilemmas in their professional work, they create these informal spaces for discussion either in-person or using social networks. Each interpreter decides whether or not to accept the recommendations given in these groups. Despite this, there are divisions among members of the D/deaf community causing miscommunication and a poorly cohesive community, resulting in knowledge dissemination difficulties.

…there is a committee formed by sign language linguists, there are certain leaders, but the same issues persist. In other words, each one forms his own “parche” [*Parche* it is a is a word used in Colombia to refer to a group of friends or close people who share ideologies, preferences or a common activity at a specific time] and sadly there is not much sharing because there is a lot of distrust between the D/deaf community and the community of interpreters. (Female 15)

### Professionalism

Interpreters need specialised skills and knowledge tailored to the specific context of each interpretation. In medical settings, ideally, they must be proficient in both the subject matter and the specific medical terminology in the CSL. Adequate preparation is essential for different types of consultation. Interpreters must maintain objectivity and ensure accurate information transmission while being mindful of time constraints during consultations.

An essential component of interpreters’ professional ethics in the healthcare context is maintaining neutrality throughout their service. Neutrality can be understood from two perspectives. First, it involves fidelity in accurately conveying information, ensuring that the intended message is effectively communicated. Second, it entails upholding professional neutrality, especially when providing interpretation for family members or friends, as interpreters are compelled to remain advocates. They must refrain from expressing personal opinions or feelings during the medical appointment. Maintaining such emotional control can be challenging, particularly when they encounter unjust situations or negative attitudes towards the D/deaf individual.

It is not fair that news makes me want to cry, and I have to hold back the urge to cry because I am interpreting […] repressing those emotions is a very, very hard thing to do. (Male 12)

…I see the gesture of the doctor, I feel the pressure on his voice[…] that day, with his gestural expressions, he did like this (bang on the table), Imagine that! So, I experienced everything. (Female 02)

Two interpreters mentioned that it is common, particularly at the beginning of a session, for the interpreter to specify to both parties that all information will be conveyed and the interpreter will not interfere in the communicative exchange. The remaining interviewees however mentioned that they never have time to prepare for a medical interpretation service and that it is difficult because they never know what patient concern will be addressed during the doctor’s appointment. For some of them, it is a deciding factor as to whether to accept an assignment or accept an interpretation job, because they often feel unqualified and it is necessary to ensure that the information is understood, especially in a healthcare context.

If there is something that the D/deaf should not know, then don’t tell me. Because if you [the doctor] tell me, I will pass it on in sign language […] they [the D/deaf patients] are already warned about what I am going to do in the interpreting service. There is no longer a personal link with me. The link that the doctor has to have is with the D/deaf person, and the D/deaf person with the doctor. (Male 17)

I always talked to her [the D/deaf patient] for a long time before going to the appointment and for a long time after, because I thought it was very delicate that she didn’t understand how often she had to take a medicine or something like that. Because I said, “oh no, something is going to happen with the baby and it will be my responsibility”. (Male 13)

All interpreters interviewed mentioned that during their training, no specific education was provided on medical/health interpretation. They state that they are told about the confidentiality of the information that must be handled in all contexts, but no training was provided about how to carry out interpreting services in the health context. Added to this, interpreters encountered several major challenges in professional interpreting that limit proper interpretation. These include lack of signs for some medical terms, duration of medical appointments (which in Colombia generally last only 15–20 min per patient), and the absence of culturally and linguistically appropriate questionnaires or tests provided by the doctor in sign language.

In the health context the formation is more general, like a training process in general terms. It is there when one as an interpreter, in many times feels like: What does this mean? … how is it transmitted? what is it? and what are the processes? what are the protocols regarding this topic? because they are completely unknown. (Male 07)

Some interpreters mentioned that D/deaf people feel more comfortable with a same-gender interpreter; however, others mentioned that D/deaf individuals feel better with interpreters of the opposite gender.

Women are a little more reserved because I am a man, so I ask many questions to get them to really say what is happening to them. And in the case of men, they are more open-minded, “take off their clothes” and they start undressing right away, they don’t have that difficulty. (Male 13)

### Ethical framework

A description of the core components that should guide sign language interpreters’ decision-making can be found in online supplemental material. The ethical framework is intended to discuss, review and encourage reflection on ethical concerns of important issues, and contribute in a meaningful way to the growing field of interpreter ethics. Based on our findings and on the literature, we propose ethical principles for sign language interpreting to provide a set of guidelines for practical decision-making that complement, not supplement, other normative approaches.

## Discussion

The empirical findings from this study allowed us to better understand the experiences of sign language interpreters working in healthcare settings. We identified key challenges and core components relating to the ethical issues experienced by sign language interpreters in Colombia, including confidentiality and privacy, professionalism, business practices and professional development. Interpreters were often the bridge between D/deaf and hearing communities. To overcome communication asymmetries, these dynamics often placed sign language interpreters in positions of uneven power dynamics and, at times, challenging social expectations. The findings from this research enabled us to introduce a framework that provides a roadmap for interpreters’ ethical behaviour and facilitates decision-making in the healthcare context.

Confidentiality is highly valued in the D/deaf community.^31^ This is particularly true in the healthcare context where sensitive information is handled. Either in-person or via VRI, D/deaf people are often concerned that the information provided during healthcare appointments will be disclosed. In addition to the universal right to privacy that all patients expect, an important cause of this concern is that the D/deaf community is a linguistic and cultural minority in which the members generally know each other.^38^ Comparing VRI with face-to-face interpretation, the interpreters interviewed in this study reported that some D/deaf clients prefer to use interpreters they know and trust and who are already familiar with the signs with which they communicate, while others prefer an unknown interpreter to guarantee confidentiality.

Because no interpreters in Colombia are currently trained in medical interpretation, some interpreters reported they were unclear about how to act in situations where a person’s privacy is compromised. Roberson and Shaw^34^ mentioned that in a medical situation, it may be appropriate not to be with the D/deaf person throughout the entire interview, especially during the physical examination or when the doctor asks the patient to remove his clothes. In such cases, the interpreter must ensure that the D/deaf person understands the instructions of the healthcare professional and what is going to happen during the procedure before exiting the exam room and when they should return to the room so their client is not left without an interpreter.^34^ Medical examinations represent a challenge; the interpreter must have visual contact with the D/deaf person, at the same time not invade their privacy, and guarantee proper comprehension of the doctor’s instructions (position or location).^39^ This is why the previous agreements made between the interpreter and the D/deaf person are essential to guarantee adequate interpretation during a medical consultation, while ensuring the patient’s privacy.

According to the NAD-RID code of professional conduct, interpreters must perform their business practices in appropriate working conditions to carry out their work effectively.^31^ In Colombia, there are no regulations for hiring interpreters; most of the institutions and entities that hire interpreters do so without looking into their professional qualifications. Many of these interpreters have only limited sign language skills, insufficient to allow them to competently interpret.^21^ This, in part, may be due to the lack of enough professional education and certification. For complex medical interpretation, interpreters decide whether to accept the service based on economic needs, despite their limitation qualification in that context.

Several interpreters interviewed reported that a significant portion of the sign language interpreting workforce in Colombia is affiliated with the Jehovah’s Witnesses denomination, a religious group known for its traditional beliefs and distinct perspectives on medical practices, such as blood transfusion.^40 41^ Participants also expressed concerns that this group has reportedly made efforts to maintain records of the D/deaf population in Colombia. Given the limited governance and regulation of interpretation services, as well as the lack of formal training for interpreters, it is important to examine whether and how the personal or institutional values of interpreters may influence communication about medical discussions and treatments for D/deaf clients.

This was reflected in our series of interviews. Most interpreters interviewed mentioned that they were introduced to sign language through their church. They receive their training through their church and prepare to assist those in need. Over the years, they have created personal ties with the D/deaf community. Due to the precarious economic situation in their profession, it is very problematic for interpreters to perform free services. Although they enter their profession for altruistic reasons, it is usually their main economic activity. The economic precariousness of some D/deaf people may lead them to ask for favours from interpreters for unpaid services, and the economic and social situation of some interpreters may lead them to offer favours and accept any job offered.

It has been observed that new signs are constantly evolving, as the D/deaf community creates new signs to keep up with new terminologies.^42^ For interpreters to maintain their professional competency, they must stay up-to-date with these new terms and phrases, as well as the latest trends in the community.^43^ This becomes particularly crucial in specialised interpreting contexts like healthcare. During this research, it was noted that interpreters reach consensus on new linguistic terms through two primary methods: expert consensus, achieved through meetings with linguistic experts, and informal consensus, which involves discussions with colleagues, discussions with community members or interactions on social networks. Sign languages evolve like any other language, with variations that persist due to a lack of standardisation, differing across geographic regions, institutions or specific contexts. That is why some sort of system to ensure consistent interpretation is much needed especially from the context of medical accuracy.

Beyond staying updated, a key aspect of achieving adequate professional development is establishing continuous communication between interpreters and the D/deaf community.^17^ In this research, the interpreters also made it clear that it is important to look for opportunities for discussion, particularly among fellow interpreters, to address ethical dilemmas they encounter. However, a significant challenge in accomplishing this lies in existing rivalries between the D/deaf community and the interpreting community. Due to lack of confidence that the interpreter is transmitting the correct information, this can hinder proper communication and feedback on their professional work. That is why we suggest starting by establishing a shared baseline to build upon, grounded in evidence. Much work is needed in legislation and policy to regain the trust of the D/deaf community.

Closeness is an issue in Colombia. Establishing professional boundaries between interpreters and the D/deaf population is crucial. Our findings correlate with the international literature which finds that interpreters often develop close relationships with members of the D/deaf community.^44^,^46^ However, this closeness may hinder their ability to maintain emotional distance and objectivity, making it more difficult to maintain confidentiality. In addition, interpreters’ personal biases and judgments can influence the interpretation process.

Our results are similar to the findings of Hommes *et al*;^47^ one of the goals for an interpreter is to be a communication facilitator, and in doing so, they may contribute to D/deaf person’s ability to exercise their rights. Interpreters must encourage the D/deaf person to actively communicate with the doctor.^47^ This empowerment is achieved by maintaining a neutral relationship with the D/deaf person, accurately conveying information, ensuring that the intended message is effectively communicated and refraining from expressing personal opinions or feelings.

Interpreters face another challenge when interpreting for medical consultations for family or friends, as they often feel compelled to take on an advocacy role but may fail to transmit the information as faithfully as possible as this situation creates constant tension in balancing their roles as members of the D/deaf community, as professionals and as a friend or family member. Therefore, ideally, it would be of paramount importance to avoid providing interpreting services to individuals with close personal ties. Instead, they should refer them to a colleague to prevent conflicts of interest and ensure optimal care for all parties involved. But, the authors recognise that, realistically, this is often hard to do.

In addition to having adequate preparation for interpreting in healthcare contexts, the results show that an additional aspect to consider in the professionalism can be the interpreter’s gender in some types of consultation (eg, sexual and reproductive health). Agreements and boundaries established prior to a medical appointment between D/deaf individuals and the interpreter would help to decrease such uncomfortable intimate situations.

A number of countries have established specific codes of ethics for healthcare interpretation in spoken languages.^30 35 48^ Unlike our research, they included other components such as maintaining ties with relevant professional organisations in order to be up-to-date with the latest professional standards and protocols, cultural responsiveness,^30^ or demonstrating respect for consumers, colleagues, interns and students of the profession.^31^ There is still disagreement on the best way to proceed in many situations and much more in sign language interpreters where there is no specific code of ethics in the health context. This is why developing an applied ethics framework for sign language interpreters in Colombia upon which to make ethical decisions and provide guidelines and principles that help both interpreters and their Deaf clients navigate complex ethical dilemmas in providing interpretation in the healthcare setting. We hope this paper has provided some background upon which a fuller and more nuanced discussion of sign language interpretation in healthcare settings in Colombia can be built.

This study has certain methodological limitations. By using interviews as the sole methodology, this study offers an initial understanding of the research topic; however, further studies employing diverse methods are recommended to gain a more comprehensive and conclusive perspective.

The study is limited to the Colombian context, which may have unique cultural, legal and professional dynamics. It is essential to replicate the study in diverse countries, because the tensions unveiled in one location may differ from those in another. We were able to interview many sign language interpreters from the country’s capital city, and a small subset of interpreters from other regions of Colombia. The framework we propose can serve as a reference in concrete cases that may be challenges for interpreters. Promoting a solutions-oriented approach to ethical analysis entails going beyond merely identifying problematic situations to proposing solutions.

Government in consultation with national Deaf organisations should take the lead in establishing and enforcing a professional accreditation system that would ensure the highest level of accurate service and privacy in medical services for D/deaf individuals throughout Colombia. Policymakers should be aware of the significant impact that interpreters have on the health outcomes of D/deaf people. If an interpreter lacks adequate training in the health context and yet institutions are forced to contract for their services, this directly impacts the health of D/deaf people. Lack of adequate training can prevent them from accessing proper education and information on the prevention and treatment of their illnesses affecting the quality of the interpreting services they receive. Therefore, it is crucial to ensure that interpreters in the healthcare setting are adequately trained, supported and prepared to provide effective and quality support to D/deaf people in their healthcare.

## supplementary material

10.1136/bmjopen-2024-086490 online supplemental file 1

## Data Availability

Data are available upon reasonable request. Data may be obtained from a third party and are not publicly available. All data relevant to the study are included in the article or uploaded as supplementary information.
