# Supplementary material for: Key components in the professional ethics of sign language interpreters in healthcare contexts: a qualitative study in Colombia
Source: BMJ Open. 2025 Feb 11;15(2):e086490. doi: 10.1136/bmjopen-2024-086490 (PMC11815455; doi:10.1136/bmjopen-2024-086490)
Supplement: online supplemental file 1 [file bmjopen-15-2-s001.docx]

| **Supplementary material.** Ethical framework to guide decision-making for Colombian sign language interpreters. |
| --- |

| **THEMES** | **POINTS OF TENSION** | **TENETS** | **EXAMPLES ILLUSTRATIVE BEHAVIOURS** |
| --- | --- | --- | --- |
| **Confidentiality and privacy** | *Maintain confidentiality of information, including when interpretation with friends or family members.  *Discomfort in privacy situations  *Providing interpretation services for family or friends  *Tension between belonging to D/deaf community, being a friend or family member and their professional work *Be completely impartial in the communicative role | *Interpreters must ensure D/deaf confidentiality and privacy (except when required by law or patient consent to share information) in healthcare context  * Interpreters must maintain the boundaries of the professional role in healthcare context | *As far as possible, an interpreter should avoid providing interpreting services to a family member, friend, or close relative. * In healthcare encounter they must refrain from disclosing any personal or sensitive information learned during their services. An interpreter must not discuss a patient’s case with family or community members without the patient’s consent. * Interpreters should respect privacy and reach a prior agreement before the health encounter with the D/deaf person on whether he/she wants the interpreter to be present in private situations.  * Interpreters will refrain from accepting an assignment when family or close personal relationships can affect their role. *The interpreter should promote confidentiality practices, patient autonomy and empowerment ensuring that the D/deaf person is always the protagonist in the communicative exchange. *The interpreters should promote direct communication between the health professional and the D/deaf *The interpreter does not allow personal judgments or cultural or religious values to influence objectivity. They will not reveal personal feelings or thoughts. |
| **Business practices** | * Poor labor legislation and regulations; lack of education and training in interpretation in specific contexts * Providing/ being called upon to provide free care for family and friends puts the interpreter in a difficult position  * Lack of laws that govern and allow enforcement of hiring agreements regarding payment | *Interpreters must maintain ethical business practices in healthcare context | * Interpreters will refrain from accepting assignments beyond their professional skills and language fluency training. They must disclose skill limitations with respect to assignments in healthcare context, especially when they are inexperienced or unfamiliar with the vocabulary used in the healthcare context. * Interpreters should ensure impartiality and avoid situations in which their personal or financial interests may influence the way they charge fees or provide services.  * The interpreter should advocate for appropriate working conditions that support quality services. They should reserve the option to decline or discontinue a service if working conditions are not safe or healthy. * They should charge fair and reasonable fees for interpretation services and arrange payment in a professional manner. * Interpreters must accurately represent qualifications, such as certification, educational background, and experience. |
| **Professional development** | * Continuous updating and learning  * Absence of prior agreements on signs  *Dialectal variations  * Lack of standardization of the signs  * Division between members of the community | *Interpreters must engage in professional development in healthcare context. | * Interpreters will keep abreast of evolving sign language and medical terminology. * Interpreters will participate in continuing education programs and will seek to maintain contact with D/deaf associations or non-governmental organizations in order to be up-to-date.  *In order to promote appropriate dissemination of knowledge, interpreters must maintain clear and respectful communication with colleagues and members of the D/deaf community. * When there is no sign for medical terms used or there are dialectal variations, the interpreters must reach prior agreements with the D/deaf person on the signs to be used during medical consultation  * The interpreter should rely on his peers or colleagues when doubts arise to improve his performance. |
| **Professionalism** | *Maintaining neutrality  *Prepare the interpretation service  *Lack of training in healthcare contexts  *Impact of the interpreter's gender on the comfort of the D/deaf person in clincial situations. | *Interpreters must always act in a professional and ethical manner. | *Interpreters will not give medical advice  *Interpreters will use skillful unobtrusive interventions without interfering  *The interpreter will disclose potential conflicts of interest, withdrawing from service if necessary.  *The interpreters will select the most suitable signs language level for the D/deaf person receiving interpretation services  *Interpreters will seek to prepare the service. Means that interpreters take proactive steps to ensure that they are ready to provide effective interpretation for a particular assignment or context. The interpreter will ask about the nature of the appointment and reviews relevant terminology prior to the time of the appointment  * The interpreter will advise parties that everything said will be interpreted. They will translate everything that is said even if it is rude or irrelevant |
