# Supplementary material for: Key components in the professional ethics of sign language interpreters in healthcare contexts: a qualitative study in Colombia
Source: BMJ Open. 2025 Feb 11;15(2):e086490. doi: 10.1136/bmjopen-2024-086490 (PMC11815455; doi:10.1136/bmjopen-2024-086490)
Supplement: Supplementary file 2 [file bmjopen-2024-086490.reviewer_comments.pdf]

PEER REVIEW HISTORY

BMJ Open publishes all reviews undertaken for accepted manuscripts. Reviewers are asked to complete a checklist review form and are provided with free text boxes to elaborate on their assessment. These free text comments are reproduced below.

ARTICLE DETAILS

Title (Provisional)

KEY COMPONENTS IN THE PROFESSIONAL ETHICS OF SIGN LANGUAGE INTERPRETERS IN HEALTHCARE CONTEXTS: A QUALITATIVE STUDY IN COLOMBIA

Authors

Izquierdo Martínez, Laura Catalina; Groce, Nora Ellen; Hurst, Samia; Martínez-R, Angela; Cuculick, Jessica; Rivas Velarde, Minerva C

VERSION 1 - REVIEW

**Reviewer**	**1**
**Name**	**Masuku, Khetsiwe P**
**Affiliation**	**University of the Witwatersrand Johannesburg, Speech Language Pathology**
**Date**	**23-Apr-2024**
**COI**	**I have no competing interests**

Thank you so much awarding me the opportunity to review this manuscript. Even though the manuscript addresses a valuable topic that has implications for access to healthcare for persons who are D/deaf, it unfortunately can't be accepted for publication in its current form. I have detailed my comments on the attached PdF document. Overall the document presents with grammatical and editorial errors. I would strongly suggest sending it to an editor. I think that the authors have argued well for the study, however it should be explained why a proposed framework is necessary, especially because in the background it is mentioned that some frameworks exist. Explain why this one is unique. The literature review section turns to gravitate more towards the context where the study is situated. Comparisons needs to be drawn to developed countries and also Sub Saharan Africa. Overall the methodology is sound, however, clarity regarding the steering committee and their role is necessary. The recruitment process for participants needs to be explained and so should participant demographics. Reference is made to figure 1, but there is no figure 1 in the document. Trustworthiness should be accounted for in the study. It is mentioned that interviews were conducted in Spanish. The

translation process of the interviews should be mentioned. How was rigour ensured in the process? How was it ensured that no meaning was lost during the translations?

The reviewer provided a marked copy with additional comments. Please contact the publisher for full details.

**Reviewer**	**2**
**Name**	**Hulme, Celia**
**Affiliation**	**The University of Manchester School of Health Sciences**
**Date**	**07-Jul-2024**
**COI**	**I declare I have no competing interests.**

It is always good to see new additions to literature regarding Deaf people and sign language interpretation. The manuscript presents a comprehensive and well-structured examination of the professional ethics of sign language interpreters in healthcare contexts, with a specific focus on Colombia. While the overall structure and content of the manuscript are strong, there are several areas that could benefit from revision to enhance clarity, consistency and impact. Please note that the comments below are not exhaustive of where areas can be revised, and further comments can be found in PDF document.

• Some further discussion is needed on ethical considerations, particularly why lacking professional boundaries is unethical, with examples of unethical practices by interpreters.

• The manuscript should be thoroughly checked for spelling accuracy.

• Some statements should be verified for truth and feasibility, ensuring all claims are supported by evidence, examples, or references.

• The methods section needs revising and reorganising. Certain sentences should be moved to the start of the methods, recruitment or ethics section to improve the logical flow and organisation of the manuscript.

• A transparent and clear recruitment section is required. The authors stated that access to a list for recruitment was via a VRI platform. Who shared the recruitment materials? How did they do it? How did participants contact the research team?

• A more detailed description of why the authors uses COREQ in the analysis section.

• Specific figures for female participants should be provided to enhance data comprehensiveness.

• The grounded theory framework needs further expansion to explain to the reader what it is.

• There needs to be more depth to the public involvement section by adding who the participants are and if there were any deaf individuals involved and how they were used in the study. If they were involved, how did you ensure information accessibility?

• The proposed ethic guidelines table was not very easy to read (no clear space between three columns, double spacing). Please ensure that it is in an easy to read format.

• It would be beneficial to include a positionality statement in the methods section about the authors backgrounds, communication methods, involvement with the Deaf community and interest in this study.

By addressing these areas, the authors can further strengthen an already valuable contribution to the field of sign language interpretation in healthcare contexts, particularly in LMICs.

The reviewer provided a marked copy with additional comments. Please contact the publisher for full details.

**Reviewer**	**3**
**Name**	**Roman, Gretchen**
**Affiliation**	**University of Rochester, Department of Family Medicine Research**
**Date**	**16-Jul-2024**
**COI**	**This reviewer has no competing interests to report.**

Thank you for the opportunity to read this interesting manuscript on the practice of medical sign language interpreting in low and middle-income countries (LMICs); specifically, in Columbia. Please refer to the supplemental file for detailed feedback.

General summary
Thank you for the opportunity to read this interesting manuscript on the practice of medical sign language interpreting in low and middle-income countries (LMICs); specifically, in Columbia. Unfortunately, many minor edits throughout the manuscript distracted this reviewer from appreciating its overall impact on and potential contributions to the literature base. In effort to strengthen the reader's focus on the content of the paper versus edits to the writing, detailed editorial feedback from this reviewer is offered below. This reviewer has tried to elevate the most pressing comments to the beginning of each section of this review report, otherwise the extensive editorial feedback can be found under "minor edits."

Abstract
The abstract was unstructured at 147 words. From the BMJ Open Author Guidelines, it states that the abstract should be structured, including (when appropriate) objectives, design, setting, participants, interventions, primary and secondary outcome measures, results, conclusions, and where applicable, trial registration. On a cursory glance, this reviewer was unable to find a word count for the abstract.

Author Guidelines can be found here: https://bmjopen.bmj.com/pages/authors#research

Introduction
The acronym VRI is for video remote interpreting, not video relay interpreting. The acronym for video relay service interpreting is VRS interpreting. In this context, this reviewer believes the authors mean VRI but is not sure.

"Sign language interpreters serve as mediators between D/deaf people and the hearing community…" This reviewer is not sure that "mediators" is the best descriptor here. Mediators play a role when there is a point of contention between two parties. Because, in the first paragraph, the authors describe the role of interpreters as "…acting as a bridge between both languages and cultures…", how about using something more along the lines of "message clarifiers" or "cultural brokers" to describe the role of interpreters?

"…with standardized training systems and defined levels of interpretation…"? This reviewer believed the authors mean certification here. There should also be a comma after either interpretation or certification to indicate the transition. This reviewer is not aware of any "increased levels of qualification based on the number of years of experience" in the United States. Levels of qualification in the United States are entirely based on skillset. Is this the case in the United Kingdom? If so, the way the sentence reads now indicates that it is the case in both countries. Please revise.

Line 9 on page 4: "Although there are already international frameworks in place that promotes a coherent…" should be "Although there are already international frameworks in place that promote a coherent…" At this point, the reader is only aware of the authors mentioning one international framework, the one that was created by NAD-RID. Why in the last paragraph of the Introduction are the authors stating "frameworks"? If there are other frameworks, can the authors also talk about them in the second to last paragraph or simply change "frameworks" to "framework" and keep "promotes" plural.

In the last couple of paragraphs in the Introduction, there needs to be a clearer transition from the training, qualifications, and interpreting certifications not available/available in various countries to the promoting the reader's awareness of the international code of professional standard of conduct created by NAD-RID and how countries without their own codes are not compliant with NAD-RID's code.

When stating the purpose of the paper in the last paragraph of the Introduction, it is not clear why the authors feel the need to provide another framework when the NAD-RID international framework is already available. Can the authors explain to the reader why a specific framework contextualized to Columbia is necessary, rather then just somehow having the medical sign language interpreters in Columbia adhere to the NAD-RID international code. This will help to further justify the need for this study.

Minor edits in the Introduction

In the first paragraph of the Introduction, deaf is first introduced with a lower case ‘d,’ then later introduced as both upper and lower case ‘D/d.’ Be consistent throughout the entire paper.

The sentence, “…deaf persons are at risk of missing out essential health information…’ should instead read, “…deaf persons are at risk of missing essential health information…”

“Low and Middle-Income Countries (LMIC)” does not need to be capitalized in advance to establishing the acronym. “High-Income Countries” does not need to be capitalized either.

“…availability of interpreters and technological tools like Video Relay Interpreting (VRI), are important…” the comma here should come after tools and not after (VRI).

Establish the UN acronym. For example, “..embedded on the United Nations (UN) Convention on the Right of Persons with Disabilities (CRPD)…” or ..embedded on the United Nations Convention on the Right of Persons with Disabilities (UN CRPD)…

“Yet in many LMIC such as those in Latin America…” should read, “Yet in many LMIC, such as those in Latin America…”

When writing about the number of interpreters in Mexico, can you add, “…there are only 42 certified sign language interpreters **in the entire country**.”?

The sentence beginning with “Training for those who function as interpreters for D/deaf people in medical settings…” is far too long and not clear. Please revise.

The following sentence needs editing, “…recognized by an official or governmental educational institution let alone medical interpretation available in many countries, and there are no official courses of studies or qualifying exams.” Consider splitting this up and beginning with, “In many countries, medical interpretation is not available. There are no official degrees in interpretation endorsed… educational institution, nor are there any official course of studies or qualifying exams.”

Line 15 on page 3: not sure that Sign Language Interpreters needs to be capitalized.

“The American National Association for the Deaf-Registry…” would better read as “The National Association for the Deaf-Registry… (NAD-RID) in the United States has created the most…” Also, this sentence is too long. Please break up.

The following sentence needs editing for clarity, “…interpretation in health care for spoken languages…”. Instead consider, “…interpretation in health care from professional associations of spoken language interpreters, such as the National Council on…”

“…for medical interpreter’s work are…” should be “…for medical interpreters’ work are…” or “…for a medical interpreter’s work are…”

This reviewer believes there should be a comma before “such as” in line 50 on page 3. After “professionalism,” this reviewer suggests a comma instead of a semicolon.

The following sentence is not clear, “Specifically, interpreters in healthcare should reference and align with those established for hearing interpreters.” Based on the preceding information in this paragraph, do the authors mean spoken language interpreters here instead of hearing interpreters? If so, please consider “Specifically, medical sign language interpreters in healthcare should reference and align with those established for spoken language interpreters.”

In the last sentence of the Introduction, do the authors mean, “…may also be relevant to other LMICs **seeking to** establish a framework…”?

Background

Sign language interpreters formation

After sharing about the recent establishment of professional level training programs in Columbia, the authors mention that most interpreters are trained by different D/deaf associations or local or national non-governmental organizations. Should the topic sentence for the second paragraph of this sub-sections read, “Prior to the establishment of these professional level training programs, most interpreters were trained by different…” This reviewer is wondering why interpreters are still being trained by D/deaf associations after the new programs were established?

In line 39 on page 5, is social media the best approach to keeping up to date on emerging signs for medical terminology?

Minor edits in Sign language interpreters formation

“Today” and “now” are redundant in the following sentence, “Today professional level training for sign language interpretation is now offered…”

“…competency and formally awarding accreditation for those who attend these programs.” Programs are accredited and interpreters work to get certified. This might be better stated as, “…competency and formally awarding accreditation for these programs and certification for those who attend them.”

“…regulations defining what agencies or groups should guarantee the quality of interpreters’ education, interpreting services” is not clear. Might better read as, “…regulations defining what agencies or groups should **do to** guarantee the quality of interpreters’ education and their skill level when providing interpreting services.”

Would add “…met any established professional **interpreting** standards.”

Not sure that a semi-colon is best served after “In a healthcare context; in theory, interpreters…” Consider “In a healthcare context, in theory, interpreters…” instead.

Consider contextualizing “D/deaf person (30,31)” in lines 22-23 on page 5 to “Deaf/deaf patient (30,31)” because the authors are discussing a healthcare setting.

This reviewer continues to not be a fan of the repeated use of semi-colons. Please edit, "The responsibility to decide; they are competent to provide accurate medical interpretation and accept a job lies in the interpreters themselves." Consider, "Interpreters have the responsibility to use their professional judgement and decide, do they have the competence to provide accurate medical interpretation and should they accept an interpreting assignment within the healthcare context?"

"A lack of knowledge of these issues, or insufficient breadth interpreters' lexicon, can heave…" might be better stated as, "A lack of **awareness** of these issues or **interpreters'** insufficient breadth **of** lexicon, can heave…"

A comma is needed after "disabilities" and before "as well as" in lines 46-67 on page 5.

Drop the semi-colon after "time" and start a new sentence, "…as well as lead to increased appointment time. If the interpretation is not going well, a visit may be cut short and the D/deaf patient will be provided less information or support than they may need."

Just like Sign Language Interpreters, not sure that Interpreters and Child of Deaf Adult need to be capitalized in line 54 on page 5.

.

Should read, "households, or members of the Jehovah's Witness religion."

Comma is needed after "…and thus, have served as interpreters…" in lines 8-9 on page 6.

Types of sign language interpretation

Same comments here as previous about use of the VRI acronym for video relay service interpreting. In the United States, video relay service interpreting (VRS interpreting) is a three-way connection over the telephone. All parties are in separate locations. Video remote interpreting (VRI) is either on-demand or pre-scheduled and is a two-way connection between onsite Deaf and hearing participants with a remote interpreter in a separate location. Please be clear about which type of interpreting is being discussed and update your acronym use throughout the paper, if needed. VRI interpreters may work in VRS interpreting centers. It is not clear from your description in this section if Columbia is offering VRI or VRS interpreting or both. Please clarify; particularly, if you anticipate readers from within the United States.

In the first paragraph of the next section (lines 17-18 on page 7), the authors describe, "…awaiting calls to provide their services." This seems like it could be VRS interpreting, but may also be on-demand VRI. Still not clear if it is one or the other or both.

When the authors state, "In most cases, with in-person interpreting, the D/deaf person must pay for this service using their own money." This is distinctly different from the United States. Possibly consider stating, "Unlike in the United States where interpreting services are available to D/deaf communities at no charge under the American with Disabilities Act, in Columbia and other countries, the D/deaf person must pay for in-person interpreting services using their own money. Although this is the case only for in-person interpreting, as remote interpreting is supposed to be free."

Hiring characteristics in the healthcare context
Instead of "…means that some interpreters provide much better services than others," can the authors indicate there is variability in the skillset of interpreters because of the inadequate training and regulation? Then, leave the good or bad distinguisher in the next sentence.

Might consider moving the "Establishing criteria to allow…", as well as the last paragraph of this sub-section beginning with "The absence of public policy…" (lines 4 – 25 on page 8) to the summary paragraph of the Introduction. This will help to provide more rationale for the study (this reviewer thought it was missing but it comes well into the paper) up front. The summary paragraph of the Introduction should provide the reader an idea of why the paper is important and a roadmap for where it plans to take reader.

<u>Minor edits in Hiring characteristics in the healthcare context</u>
"Some interpreters are hired by D/deaf clients**, while** others are hired and work…"

Please edit this sentence "A few are hired and kept on call by health care (health care or healthcare, please keep the same throughout the entire paper) facilities, which; choose to incorporate…" Consider "A few are hired and work on call for a hospital or clinic that elects to provide interpreting services within their facility."

"Possibly, some interpreters work for all of **these entities**."

No need for the "However" transition. Begin with "Interpreters **also** provide their services…"

"Favor" spelled two different ways, favor and favour," in lines 26 and 50 on page 7.

Material and methods
If appropriate for the journal, this reviewer would like to see a positionality statement from the authors. Based on author affiliations, the authors are researchers in Switzerland, Argentina, England, and the United States. This reviewer feels it would be helpful for the readers to understand how the researchers are affiliated with and aware of sign language interpreting in Columbia, specifically. Also, it would be helpful to know if any of the authors are sign language interpreters themselves and/or consumers of sign language interpreting services.

Citations/references for the research design and analysis based on grounded theory are needed.

The citation/reference for lines 36-38 on page 8 is from 2010. Is that citation the already published proposed framework to guide decision-making that resulted from this qualitative study? Or, was your proposed ethics framework based on guidance from that citation, as well as the semi-structured interviews? It is not clear the way it reads now. If the latter, the methodology of how it was incorporated needs to be described… coming back to this comment after reading the Data collection, this reviewer now sees that data were collected between August 2022 through February 2023. Please clarify the use of citation (35) as a component of the methodology for the reader here.

Participant involvement

Minor edits in Participant involvement

Lines 50-57 on page 8 need to be divided into two sentences. "…held regular meetings to guide the direction of the study. These meetings offered invaluable insights, provided critical feedback…"

"…and its contribution to the Deaf community, as well as disability…" need to be edited for better flow with the previous two things there were listed.

Sample

Typically, when describing the research that was conducted, it is presented in the past tense. Strike "One of the challenges of this research is that" and instead begin with "In Columbia, there is no database of professional or practicing sign language interpreters who work in healthcare settings."

"(21 were contacted and 17 agreed to participate)" should be reported in the Results section. Here, in the Material and methods, describe your inclusion and exclusion criteria, as well as any information or calculation that was used to help the authors determine sample size. No need to report the number of participants here and again, later in the Results.

Minor edits in the Sample

More editing is needed to the writing here. "Interpreters who work in-person and VRS (or what ever the authors decide with their labeling of this remote context) were selected to provide maximum variation sampling across the types of medical interpretation and geographical location."

Data Collection

When referencing software throughout technical writing, parenthetically include the (name of the company, city, state). For Zoom, this reviewer typically indicates "…using video conferencing software (Zoom, San Jose, CA)."

"…ethical issues related to exercising their profession" is not clear in lines 43-46 on page 9.

Lines 47 – 55 are explaining how data saturation was determined. Can the authors describe, "Data saturation was determined after 17 interviews when the research team considered the data sufficiently robust and suspended the recruitment." This reviewer is confused when the authors explain that data saturation was achieved after 14 interviews, but then they go on to say that 17 interviews were conducted. Possibly leave the mention of 14 interviews out, unless it can be made more clear.

Acronyms used in Table 1 need to be defined in a "Notes: LSC=; VRI interpreting=" section at the bottom of the table.

Analysis

The first two sentences of this section can be combined. "Data analysis was performed using the constant comparative method, where data collection and analysis occurred simultaneously." The comment about snowball sampling belongs in the Sample sub-section of the Material and methods section.

The portion about why video recording the interviews "was crucial" belongs in the Data collection sub-section. Consider labeling that sub-section as Data collection and processing. Try and differentiate sentences when the authors are describing collection and processing versus the actual analysis.

Provide (name of the company, city, state) for NVivo just like this reviewer has requested for Zoom.

In line 56-59 on page 10, "Every three to four interviews, the primary investigator (initials) reviewed the interpretation with the team and discussed the results." Do the authors mean the research team here, not researcher's team? If so, then should read, "…the research team (LI, MR, AM) reviewed the interpretations and discussed the results." Did the research team triangulate the results, rather than discuss?

Consider moving lines 3-10 on page 11 after the sentence about "Transcriptional coding… were organized into themes and sub-themes." Then, move "To improve intercoder reliability…" to the end. The description of the coding is split up the way it conveys now.

The Analysis sub-section describes the qualitative data analysis using the constant comparative method, but this reviewer is also interested in the grounded theory methodology the authors used to draw from the qualitative results and create the ethical framework. This can be expanded on in the first paragraph on the Material and methods section (when referencing citation 35), as well as in the Analysis sub-section. Please describe how themes and sub-themes were used to guide "Points of tension" and "Tenets," so the reader knows what to anticipate in the Results.

Results

Minor edits in Results

Spell out 4 in line 49 on page 11.

Should line 56 on page 11 read, "…in healthcare contexts"?

Confidentiality and privacy

"Mediators" is used again to reference the role of interpreters in line 34 on page 13.

The Spanish translation to English for the quotation in lines 51 – 59 on page 14 is not clear.

The Confidentiality and privacy sub-section of the Results seems long (four pages). Is there a better way to organize the Results, rather than just using the four themes that revealed the key components and core challenges? This reviewer is getting a bit lost parsing through each theme. How does being an advocate and empowering the D/deaf (lines 23 - 28 on page 15) relate to confidentiality and privacy?

Minor edits in Confidentiality and privacy

"Although most of the sign language interpreters are not trained on any medical, the idea…" The language here needs to be elevated. How about "Although most of the sign language interpreters do not have any medical training, the idea…"

"…was an important subject when they were trained…" Consider "…was an important subject during their interpreter training…"

Instead of "Interpreters reported that the D/deaf prefer to call…", how about "Interpreters reported that D/deaf patients prefer to call…"

The grammar in the participant quotations needs to be addressed. Can the authors continue to use D/deaf through the quotes? In line 26 on page 13, change the colon to a comma. "And she said to me, "What a shame that you are going to see me naked." In line 50 on page 13, "…when a doctor tells you, "take off your clothes," generally they take off all of their clothes. I said, "what do I do?..." In lines 28 – 38 on page 17, divide up the quotation into a couple of sentences.

"When a D/deaf person **is** asked to undress…"

Line 16 on page 15, "…caused them to **have** conflicts…"

"Discussed" might be better than "handled" in line 18 on page 15.

"As interpreters, they see themselves as advocates…" would be better written as "Interpreters see themselves as advocates, empowering…"

Lines 32-36 on page 15, "Communication in healthcare setting poses challenges to this role, however, as health professionals often speaks directly to the interpreter…" If this new paragraph is a continuation of the advocacy and empowerment role discussed in the previous paragraph, please combine. The sentence above needs editing, instead consider "Communication in healthcare setting poses challenges to this advocacy and empowerment. Healthcare professionals often speak directly to the interpreter…"

In lines 36 – 29 on page 15, "…they strive to uphold their empowerment role…" or "…they strive to uphold their role of empowerment…" would read a little better.

Participant quotation in lines 44 – 53 on page 15 needs to be indented. "(P13)" also needs to be edited.

Business practices
Can the authors help this reviewer understand how providing support to colleagues fits under the theme of Business practices? Can headers be created for the sub-themes? This reviewer believes the authors are talking about the altruism sub-there here?

This reviewer would like to know more about the quality team. Where do they come from? Can more context be provided here to how the quality team periodically evaluates the interpretation?

This reviewer is confused in lines 10 – 17 on page 18 when the authors are talking about "…private users hire any person who is certified to have as a sign language interpreter…" Do the authors mean "…private users hire any sign language interpreter who is certified…"? Can this paragraph be revised?

Also, this is the first time this reviewer is reading about certified sign language interpreters because from the Introduction, it did not seem there were any certified interpreters in Columbia.

<u>Minor edits in Business practices</u>
Line 51 on page 16, "However, **the lines are also blurred** regarding payment for interpreting services."

Line 7 on page 17, "…if the person with whom they are close **to** has limited financial resources."

"Due to the lack of a law regulating the working conditions for sign language interpreters…" might be better written as, "Due to the lack of legal regulations on the working conditions for sign language interpreters…"

Lines 41 - 46 on page 17 needs to be edited. "A professional concern is that VRI interpreters mentioned is concern over accuracy of that signs used." Instead consider "VRI interpreters mentioned that accuracy of the signs used is a professional concern."

"…D/deaf and hearing persons with the interpreting experience…" should read "…D/deaf and hearing persons with interpreting experience…"

Professional development
This reviewer enjoyed reading about the informal support groups for interpreters.

<u>Minor edits in Professional development</u>
"…with the individuals they are assisting" (lines15 page 19) is paternalistic. Possibly consider, "…with the D/deaf patients" or something more neutral.

"…with signs dialectal variations…" Instead, please consider "…with the dialectal variations of signs…"

In line 27 on page 19, "They face," or simply remove.

Professionalism
"…and strive to be entirely invisible…" Healthcare providers here in the United States often view the medical sign language interpreter as a member of the broader healthcare team. To presume the role of interpreters as being "entirely invisible" is not the best approach, as they are often relied upon for their professional judgement in pre- and post-sessions with the providers. Can the authors strike the following sentence, "They must refrain from expressing personal opinions or feelings…" or edit it to read, "They must refrain from expressing personal opinions or feelings during the communication exchange"?

For the quotation in lines 29 – 39 on page 21 (as well as in other quotations), it would be helpful for the reader if some of the pronouns are contextualized. For example, "…Because if you tell me, I will pass it on in sign language […] they [the Deaf patient] are already warned about what I am going to do…" Same comment for the quotation in lines 41 – 51 on page 21, "…I always talked to her [who is her?] for a long time before going to the appointment…"

Lines 47 – 52 on page 22 should go in the Analysis sub-section of the Material and methods. "Based on the results of the interviews… International Medical Interpreters Association" to create a framework that will guide decision-making for sign language interpreters in Columbia.

Here, in the Results is a new section needed to differentiate the ethical framework from the fourth theme (Professionalism)?

<u>Minor edits in Professionalism</u>
Needs to be italicized, as it is the fourth identified theme.

The "Lengua de Señas Colombiana (LSC) or the sign language of Columbia" acronym needs to be established. Line 24 page 20 should read "…specific medical terminology in Lengua de Señas Colombiana (LSC) or the sign language of Columbia."

Line 20 on page 21, "…and it is difficult because they never know the topic to be treated in the doctor's appointment" might be better stated somehow. Consider "…and that it is difficult because they never know what patient concern will be addressed during the doctor's appointment."

Does "(Male P17)" need to be corrected?

Line 56 on page 21, should be in past tense. "…no specific education **was** provided on medical/health interpretation." Same comment about past tense in line 3 page 22. "…no training **was** provided **about** how to carry out interpreting…" Again, in line 5 on page 3, "Added to this, interpreters, encountered several…" Because the authors are reporting on the Results from their study that is now complete.

"…encounter several major challenges in professional interpreting that **limit** proper interpretation."

Can "which in Columbia generally last only 15-20 minutes per patient" be placed in parentheses?

Line 32 on page 21, "…that **the** D/deaf individual…"

Discussion
The initial summary paragraph should be written in the past tense. This paragraph typically includes re-stating the study rationale and goal(s), along with a concentrated summary of the methodology and findings.

Regarding the following statement, when the authors are re-stating the goal of the study, "We identified key professional points of ethical issues of sign language interpretation…" Can the same language be used here as was used in the title, when establishing the goal in the abstract and Introduction, and when describing the results of the analysis in the Results? "We identified key challenges and core components relating to the ethical issues experienced by sign language interpreters in Columbia." This will help to streamline the paper for the reader.

“Trade-off” does not seem to be the appropriate word for describing what the authors are explaining. Instead, consider “Upon comparing VRI with face-to-face interpretation, the interpreters interviewed **in this study reported** that some D/deaf clients prefer to use…”

When the authors state, “That’s why some sort of system to ensure consistent interpretation is much needed…”, this reviewer wonders how adherence to such a system is going to be enforced?

This reviewer appreciates the use of “mediate” again in the last paragraph on page 30. Homes et al. (2018) state that the primary role of the interpreter in the healthcare system is to listen, observe, and transfer information. Again, this reviewer feels that “mediate” or “mediator” is a more appropriate term for when there is a point of contention between two parties. Please consider another descriptor.

<u>Minor edits in the Discussion</u>
Just as was mentioned for the participant quotations in the Results, please continue to use “D/deaf,” instead of deaf, here for consistency.

“The findings from this researchs allows us to introduce…” should read, “The findings from this research allow us to introduce…”

“…or VRI interpretation…” is redundant. If VRI is established as video remote interpreting, then this should read “or when VRI.” If VRI is established as video remote interpretation, then “or during VRI.”

Whenever referencing the research that was conducted, it should be presented in the past tense. In lines 9 – 11 on page 28, “…medical interpretation some interpreters **reported** they were unclear…” “Roberson and Shaw (2018) **mentioned** that in a medical situation…”

Lines 30 – 42 on page 28 create a very short (2-sentence) paragraph. Consider merging this paragraph with the previous paragraph.

Line 57 on page 28 should read, “This**,** in part, may be due…”

Line 59 on page 28 to line 3 on page 29 should read, “For complex medical interpretation**,** interpreters decide…”

Line 8 -9 on page 29, “The majority of **the** sign language **interpreting** workforce in Columbia…”

When discussing the Jehovah’s Witness faith, in the “It is known for its conservative…” sentence, there needs to be a comma before “such as” and the acronym “LGBTI” needs to be established.

Lines 20 – 23 on page 29, please add “religious” so there is no confusion about which community the authors are indicating, “…it is concerning that the values of this **religious** community may be influencing…”

“This **was** reflected in our series of interviews.” This reviewer is not sure why the authors are providing a reference for data gathered from this study when saying, “Most interpreters interviewed mentioned that they were introduced to sign language through their church **(19,30)**.”

“They acquire their training via their church, and they train to help the needy” needs editing.

“Sign languages evolve as any other language, where sign variations persist, lacking standardisation and varying according to each geographic region, institution, or specific context” needs editing. The meaning is not clear.

This is the first the authors are mentioning a rivalry between the Deaf and interpreting communities. Thank you for further explaining that the rivalry is due to the lack of confidence in the interpreter. Unfortunately, the sentence in lines 29 – 37 on page 30 is too long and needs editing. Can it be divided into 2 sentences with a period after “…between the D/deaf community and the interpreting community”?

Consider making lines 54 – 59 on page 30 two sentences.

Lines 8 – 9 on page 31 should read, “…effectively communicated**,** and **refraining** from expressing personal opinions or feelings.”

The sentence, “But realistically, this is currently often hard to do” might be better stated as, “But, these authors recognize that, realistically, this is often hard to do.”

Again, a very short (2-sentence) paragraph between lines 33 – 42 on page 31. Consider merging this paragraph with another paragraph discussing a recommendation based on similar sub-theme.

The meaning of the following sentence is not clear. “An applied ethics framework for sign language interpreters in Columbia upon which to make ethical decisions and provide guidelines and principles that help both interpreters and their Deaf clients navigate complex ethical dilemmas in providing interpretation in the healthcare setting.” Please edit.

Strengths and limitations of the study
It is nice that the authors return to what was mentioned in the Introduction here by stating that the approach to establishing an ethical framework in this study may also be relevant to other LMICs. This helps to lay the foundation for other possible future work from these authors. The solutions-oriented approach is clear and the recommendations for establishing next steps in partnership with Deaf organizations are strong.

This section starts to feel a little redundant though. Can it get pared down and some of this material instead be used to create the initial summary paragraph of the Discussion?

It is this reviewer’s understanding that the Strengths and limitations of this study section for original research should be after the abstract, rather than at the end of the paper.

Per the BMJ Open Author Guidelines, “This section should be no more than 5 bullet points relating specifically to the methods - not the results of the study. This will be published as a summary box after the abstract in the final published article.” Please revise.

Funding
None listed.

VERSION 1 - AUTHOR RESPONSE

REVIEWER: 1	ANSWER
Not just limited to information, but also Compromised quality of care; Infringement on participants’ right to confidentiality; Communication barriers negatively influenced participants’ decisions to access healthcare services	Compromised quality of care was added in Page 1
Literature for Sub saharan Africa is missing. Different picture and perspective	Interpreter’s training in South Africa was added in Page 3.
Why are these legislations not being fully complied with? Not complied with by whom? Why is a new framework a solution?	We added that these legislations are not being fully complied due to factors such as inadequate training programs,
What is being covered in this new framework that is missing from the others?	limited awareness of these standards, and the lack of formal certification programs in many regions. Page 4
Are the challenges in rural and urban similar	No. Clarification is made on the page 9
The difference between these two categories of interpreters needs to be stated earlier.	Clarification is made: Many sign language interpreters, while fluent in Colombian Sign Language (CSL), have not been specifically trained to work in specialised areas, such as healthcare, and lack knowledge of clinical care medical terminology or significant ethical concerns in a medical setting. Page 7
Explain the translation of data from Spanish to English. Who did this? How was it ensured that meaning was not lost during this process? Back translations?	The process is explained in detail in page 12
How were discrepancies addressed during coding?	We added that: After every three to four interviews, the researcher team held review sessions to examine the interpretations and discuss any discrepancies during coding and analysis of the the results (LI, MR, AM). Page 14

How was trustworthiness ensured?	We added that: Investigator triangulation during data analysis further addressed credibility and confirmability, it also ensured interpretations were not based on the first author’s understandings alone. Pag 13-14

REVIEWER: 2	ANSWER
I note that there is a definition of Deaf/deaf under (1) at the end of the document before references. I think the placement of the (1) in this paragraph is incorrect. It should be placed at the end of the paragraph. Also, I question if this should be D/deaf like the rest of the document.	A footnote is placed at the bottom of the page 1
Is there a reason for this? Supply? Cost?	Clarification is made:largely because a shortage of interpreters nationwide Pag 2
How did they become certified? It would be useful to briefly explain...	The paragraph with its explanation can be found on the page 3
What are the issues of not following professional standards? Do you have any examples of a situation where standards were not followed?	We added that: Not following professional standards in sign-language interpretation can lead to several serious issues, especially in healthcare…Pag 6

What is unethical about not having professional boundaries? Any examples of interpreters showing unethical practices that goes beyond the boundaries?	We added that: Not maintaining professional boundaries can result in several unethical practices in interpretation, such as loss of objectivity, where personal feelings or relationships interfere with impartiality and affect the accuracy of the interpretation. Pag 10
How? Any examples?	An example is added on page 9
Not sure if 'material' is necessary. Just 'Methods' will suffice	Title changed only for ‘methods’
Who are they? Were any D/deaf people involved? how did you ensure information was accessible to them? Expand more about patient and public involvement. were interpreters involved?	The information in this section is expanded. Page 11
Need a recruitment only section and mention that it was via a relay institution centre. It is important to be as transparent and detailed as possible about recruitment process. The authors mention that want to interview those who work remote and in-person. How did the authors reach those who do not work in VRI?	The information in the section recruitment and sample is expanded. Page 12
move to analysis section and please expand further why the COREQ was used - how did it guide the analysis/results?	Information expands on pages 14-15
Move to start of methods section	The section was moved pages 10-11

If possible, can the authors provide information on their backgrounds i.e. CODA, Jehovah Witness background etc?	Not possible. While the interviews aimed to understand the role of sign language interpreters, their experiences, training processes, and the challenges and facilitators they encounter in healthcare settings, exploring their personal journeys and backgrounds was beyond the scope of this research. However, we have included participant characteristics in<br>Table 2 on page 15.
It would be beneficial to give figures for the females.	The females information was added.<br>Page 14
What are the conflicts about dealing with intimate information? this is the same for any interpreter regardless of their backgrounds/beliefs	We added that: They reported that training as an interpreter based on religion caused them to have conflicts when interpreting in medical consultations where intimate information of the D/deaf person is discussed, such as cytology or urology appointments. Page 18
victims of what? I have not yet seen any evidence of them being a victim or having legal action taken against them...	This phrase was erased from the manuscript

It would impactful to continue this sentence by mentioning that despite this, they accept work that do not match their skill set rather than in a separate sentence.	We added: …despite this, many still accept positions that exceed their ability for which to provide accurate translations. Page 20
Worth reminding the reader that it is the deaf persons responsibility to pay in-person interpreting.	We added that on the text. Page 21
How is this done? Through formal routes (evaluation) or anecdotally?	All interpreters report that entities, institutions, or D/deaf individuals often hire anyone with some levels of sign language as an interpreter due to the varied quality and diversity of existing training programs. Page 23
each interpretation what? setting?	Interpreters need specialized skills and knowledge tailored to the specific context of each interpretation Pag 25
The heading needs a bit more information - ethical framework to guide decision making for whom?	The title was changed. Page 27
I found the table format difficult to read - perhaps add column lines to differentiate between the three columns.	Pag 27-33<br>Column lines added to the table.
Any suggestions on a resolution?	We added: That is why we suggest starting by establishing a shared baseline to build upon, grounded in evidence. Pag 35

I would mention that the closeness is an issue in colombia.	We added on Page 35

**REVIEWER: 3**	**ANSWER**
The acronym VRI is for video remote interpreting, not video relay interpreting. The acronym for video relay service interpreting is VRS interpreting. In this context, this reviewer believes the authors mean VRI but is not sure.	The acronym was changed on page 2
“Sign language interpreters serve as mediators between D/deaf people and the hearing community…” This reviewer is not sure that “mediators” is the best descriptor here. Mediators play a role when there is a point of contention between two parties. Because, in the first paragraph, the authors describe the role of interpreters as “…acting as a bridge between both languages and cultures…”, how about using something more along the lines of “message clarifiers” or “cultural brokers” to describe the role of interpreters?	We change the word for ‘message clarifiers’. Page 2
“Although there are already international frameworks in place that promotes a coherent…” should be “Although there are already international frameworks in place that promote a coherent…” At this point, the reader is only aware of the authors mentioning one international framework, the one that was created by NAD-RID. Why in the last paragraph of the Introduction are the authors stating “frameworks”? If there are other frameworks, can the authors also talk about them in the	Information expands on Page 4-5
second to last paragraph or simply change “frameworks” to “framework” and keep “promotes” plural.	
In the last couple of paragraphs in the Introduction, there needs to be a clearer transition from the training, qualifications, and interpreting certifications not available/available in various countries<br>to the promoting the reader’s awareness of the international code of professional standard of conduct created by NAD-RID and how countries without their own codes are not compliant with NAD-RID’s code.	Information expands on Page 4: “compliance remains limited among healthcare institutions, policymakers, associations representing the D/deaf community and interpreters. Even countries without their own codes are not compliant with NAD-RID’s code”
When stating the purpose of the paper in the last paragraph of the Introduction, it is not clear why the authors feel the need to provide another framework when the NAD-RID international framework is already available. Can the authors explain to the reader why a specific framework contextualized to Columbia is necessary, rather then just somehow having the medical sign language interpreters in Columbia adhere to the NAD-RID international code. This will help to further justify the need for this study.	Information expands: ‘Building on existing international frameworks, this Colombia-specific addresses the cultural and legislative context essential for meeting the unique needs, values, and preferences of the local D/deaf community. Additionally, it considers the variations in language, ensuring that interpretations are accurate and culturally appropriate’. Page 5

After sharing about the recent establishment of professional level training programs in Columbia, the authors mention that most interpreters are trained by different D/deaf associations or local or national non-governmental organizations. Should the topic sentence for the second paragraph of this sub-sections read, “Prior to the establishment of these professional level training programs, most interpreters were trained by different…” This reviewer is wondering why interpreters are still being trained by D/deaf associations after the new programs were established?	The sentence was changed: Page 6
In the United States, video relay service interpreting (VRS interpreting) is a threeway connection over the telephone. All parties are in separate locations. Video remote interpreting (VRI) is either on-demand or pre-scheduled and is a two-way connection between onsite Deaf and hearing participants with a remote interpreter in a separate location. Please be clear about which type of interpreting is being discussed and update your acronym use throughout the paper, if needed. VRI interpreters may work in VRS interpreting centers. It is not clear from your description in this section if Columbia is offering VRI or VRS interpreting or both. Please clarify; particularly, if you anticipate readers from within the United States.<br>In the first paragraph of the next section (lines 17-18 on page 7), the authors describe, “…awaiting calls to provide their services.” This seems like it could be VRS interpreting, but may also be on-demand VRI. Still not clear if it is one or the other or both.	The type of interpreting service referred to is Video Remote Interpreting (VRI). It is left as such throughout the manuscript

When the authors state, “In most cases, with in-person interpreting, the D/deaf person must pay for this service using their own money.” This is distinctly different from the United States. Possibly consider stating, “Unlike in the United States where interpreting services are available to D/deaf communities at no charge under the American with Disabilities Act, in Columbia and other countries, the D/deaf person must pay for in-person interpreting services using their own money. Although this is the case only for in-person interpreting, as remote interpreting is supposed to be free.”	Information expands: ‘…unlike in some high-income countries, such as the United States, where funding options are more readily available’ Page 7
If appropriate for the journal, this reviewer would like to see a positionality statement from the authors. Based on author affiliations, the authors are researchers in Switzerland, Argentina, England, and the United States. This reviewer feels it would be helpful for the readers to understand how the researchers are affiliated with and aware of sign language interpreting in Columbia, specifically. Also, it would be helpful to know if any of the authors are sign language interpreters themselves and/or consumers of sign language interpreting services.	Although we appreciate the positionality statement, please note that our methodology section, specifically under 'Patient and Public Involvement,' now includes details on how the research is governed and who is involved.

The citation/reference for lines 36-38 on page 8 is from 2010. Is that citation the already published proposed framework to guide decision-making that resulted from this qualitative study? Or, was your proposed ethics framework based on guidance from that citation, as well as the semi-structured interviews? It is not clear the way it reads now. If the latter, the methodology of how it was incorporated needs to be described… coming back to this comment after reading the Data collection, this reviewer now sees that data were collected between August 2022 through February 2023. Please clarify the use of citation (35) as a component of the methodology for the reader here.	The reference is adjusted in the text. Page 10
In line 56-59 on page 10, "Every three to four interviews, the primary investigator (initials) reviewed the interpretation with the team and discussed the results." Do the authors mean the research team here, not researcher's team? If so, then should read, "…the research team (LI, MR, AM) reviewed the interpretations and discussed the results." Did the research team triangulate the results, rather than discuss?	The paragraph has been adjusted to make the information clearer. Page 14
The Analysis sub-section describes the qualitative data analysis using the constant comparative method, but this reviewer is also interested in the grounded theory methodology the authors used to draw from the qualitative results and create the ethical framework. This can be expanded on in the first paragraph on the Material and methods section (when referencing citation 35), as well as in the Analysis sub-section. Please describe how themes and sub-themes were used to	We have clarified the methodology section to outline the approach taken and its development. Efforts have been made to ensure clarity, expand on key factors, and eliminate unnecessary details.

guide "Points of tension" and "Tenets," so the reader knows what to anticipate in the Results.	
The Confidentiality and privacy sub-section of the Results seems long (four pages). Is there a better way to organize the Results, rather than just using the four themes that revealed the key components and core challenges? This reviewer is getting a bit lost parsing through each theme. How does being an advocate and empowering the D/deaf (lines 23 - 28 on page 15) relate to confidentiality and privacy?	Each session was divided by the respective subtopics.<br>Deaf empowerment section was moved to the topic of altruism at business practices section.
This reviewer is confused in lines 10 – 17 on page 18 when the authors are talking about "…private users hire any person who is certified to have as a sign language interpreter…" Do the authors mean "…private users hire any sign language interpreter who is certified…"? Can this paragraph be revised? Also, this is the first time this reviewer is reading about certified sign language interpreters because from the Introduction, it did not seem there were any certified interpreters in Columbia.	The paragraph is revised and adjusted in page 22
this reviewer wonders how adherence to such a system is going to be enforced?	This is indeed a crucial issue, and it will require time, careful observation, and additional studies to assess whether this goal has been reached
It is this reviewer's understanding that the Strengths and limitations of this study section for original research should be after the abstract, rather than at the end of the paper. Per the BMJ Open Author Guidelines, "This section should be no more than 5 bullet points relating specifically to the methods - not the results of the study. This will be published as a summary box after the abstract in the final published article." Please revise.	Section added after the abstract

VERSION 2 - REVIEW

**Reviewer**	**2**
**Name**	**Hulme, Celia**
**Affiliation**	**The University of Manchester School of Health Sciences**
**Date**	**21-Nov-2024**
**COI**	

Thank you to the authors for the revised manuscript. It is a much more structured and specific compared to the original. I am pleased to see that the authors have addressed most of the reviewer comments.

However, there are some minor areas that need refining/clarifying before being accepted for publication.

• Abstract – The objectives, setting and participants need to be expanded more. It is a shame to see them bullet pointed. As the abstract is the main window to attract people to read the article.

• Line 9 Page 2: In Strengths and limitations of this study. You state that 'this is the first study that identifies professional ethics core components of medical sign language interpreters.' Does this apply to all in the interpreters in the world? I don't think so – I would add 'Columbia' to this statement.

• Line 9 Page 4 – 'serve as message clarifiers'. I would reconsider this explanation - 'serve' and 'message clarifiers' it is not a typical or correct way of describing the role of any type of interpreter.

• Line 11 Page 4 – 'the interpreters' impact on health outcomes…' What is their impact? Is it by providing an accurate translation?

• Line 8 Page 5 – spelling: 'nos' should be corrected

• Line 56 page 6 – spelling: 'identifie' should be corrected

• Line 37 page 8 – The misunderstandings that you list – please add references.

• Line 21 page 9 – spelling: 'heave' should be corrected

• Line 29 Page 9 – 'and discussions about their health but also make result in longer appointment times'. I would suggest changing to 'and discussions about their health and this results in longer appointment times'.

• Line 27 Page 9 – 'a visit may also be cut short'. Cut short by whom?

• Line 6 Page 10 – 'This is not unproblematic'. Is it the correct word to use? I would view what you said in this paragraph as problematic.

• Line 24 Page 10 – 'In contrast, VRI is supposed to be a free service.' I am bit confused. Is it a free service or not? As you explain below that VRI is financed by the Ministry of Information and deaf people can communicate free. You state 'VRI is supposed to be a free service'; this implies that it is not free. Perhaps wording got lost in translation?

• Line 59 – Page 13. There needs to be more information in the recruitment section. How many were recruited? How many were interviewed? You said that 17 interviews were enough for data saturation. Did you do 17 interviews? Or did you do more? What did you use for recruitment? Poster? Invitation text via email?

• Line 20 page 14 – 'to access a list' - What was included in the list? names, contact details? Phone numbers? Email address? Home address?

• Line 37 page 14 – 'informal conversations with potential participant' - should this be informal conversations with Colombian sign language interpreters not participants?

• Line 10 page 15 – ’17 interviews’. How many interviews did you offer? How many did you recruit and not offer interviews to?

• Line 46 page 29 – Ethics framework paragraph. ‘Based on findings on the literature..’ the sentence is to be swapped over with the ‘A description of the core components’ sentence as it should start the paragraph.

• I note that more than one reviewer has requested that the authors include a positionality statement. The authors have not added one and it is different from the PPIE statement in the manuscript. This should be about the authors’ backgrounds, their languages, work in sign language field etc. It adds to the credibility, validity and transparency of the work.

**Reviewer**	**3**
**Name**	**Roman, Gretchen**
**Affiliation**<br>**Research**	**University of Rochester, Department of Family Medicine**
**Date**	**04-Dec-2024**
**COI**	

Thank you to the authors' for their diligent response to this reviewer's (Reviewer 3) comments. This reviewer had the opportunity to review through the feedback she provided with the edits made by the authors and she was thoroughly impressed with their overall attention to detail. The overall clarity and presentation of the revised manuscript is much improved. The only outstanding comment related to this reviewer's interest in more expansion about the grounded theory methodology. The authors cite Jewell (2010) and mention how they aligned their findings with established international guidelines and practices. However, it is still unclear how Jewell (2010) specifically informed the conversion of the themes and sub-themes from the qualitative data into the "Points of tension" and "Tenets" of the new ethical framework for Colombian sign language interpreters. Because the authors were so clear about how the research team reached consensus and their use of the constant comparative method (they also briefly mention use of inductive analysis), this reviewer doesn't feel that this outstanding comment should hold up the progression of this manuscript's review. Best of luck with this fantastic work and the other scholarly products that are sure to result from the broader study!

VERSION 2 - AUTHOR RESPONSE

REVIEWER: 2	ANSWER
Abstract – The objectives, setting and participants need to be expanded more. It is a shame to see them bullet pointed. As the abstract is the main window to attract people to read the article	The abstract include: Objectives, design, setting, participants, results and conclusions according to instructions for authors
In Strengths and limitations of this study. You state that 'this is the first study that identifies professional ethics core components of medical sign language interpreters.' Does this apply to all in the interpreters in the world? I don't think so – I would add 'Columbia' to this statement.	All the section of Strengths and limitations was changed.
– 'serve as message clarifiers'. I would reconsider this explanation - 'serve' and 'message clarifiers' it is not a typical or correct way of describing the role of any type of interpreter.	The term was changed among all the manuscript
– 'the interpreters' impact on health outcomes…' What is their impact? Is it by providing an accurate translation?	The sentence was changed for: "Their impact on the health outcomes of D/deaf people is significant, as they improve access to healthcare information, enhance communication, enable timely and accurate diagnoses, and contribute to other key aspects of healthcare delivery"
The misunderstandings that you list – please add references	Reference added
'This is not unproblematic'. Is it the correct word to use? I would view what you said in this paragraph as problematic.	The word was changed for 'This presents challenges…'
'In contrast, VRI is supposed to be a free service.' I am bit confused. Is it a free service or not? As you explain below that VRI is financed by the Ministry of Information and deaf people can communicate free. You state 'VRI is supposed to be a free service'; this implies that it is not free. Perhaps wording got lost in translation?	The VRI service itself is indeed free for Deaf individuals, as it is financed by the Ministry of Information. However, Deaf people are required to use their own phones and internet to access the service, which means they may still incur personal costs related to data or phone usage. So, while the service is free in terms of the actual communication, there are potential indirect costs that make it not entirely without expense.
There needs to be more information in the recruitment section. How many were recruited? How many were interviewed? You said that 17 interviews were enough for data saturation. Did you do 17 interviews? Or did you do more? What did you use for recruitment? Poster? Invitation text via email?<br>'to access a list' - What was included in the list? names, contact details? Phone numbers? Email address? Home address?<br>'17 interviews'. How many interviews did you offer? How many did you recruit and not offer interviews to?	The entire recruitment and sampling section was revised to provide more detailed information. Pag 14

I note that more than one reviewer has requested that the authors include a positionality statement. The authors have not added one and it is different from the PPIE statement in the manuscript. This should be about the authors' backgrounds, their languages, work in sign language field etc. It adds to the credibility, validity and transparency of the work.	Positionality statement paragraph was added on the methods section

REVIEWER: 3	ANSWER
The only outstanding comment related to this reviewer's interest in more expansion about the grounded theory methodology. The authors cite Jewell (2010) and mention how they aligned their findings with established international guidelines and practices. However, it is still unclear how Jewell (2010) specifically informed the conversion of the themes and sub-themes from the qualitative data into the "Points of tension" and "Tenets" of the new ethical framework for Colombian sign language interpreters. Because the authors were so clear about how the research team reached consensus and their use of the constant comparative method (they also briefly mention use of inductive analysis), this reviewer doesn't feel that this outstanding comment should hold up the progression of this manuscript's review.	We understand the concern regarding potential reader confusion. As the analysis was conducted inductively, we used Jewell's (2010) work primarily as a reference to appropriately name the codes. To avoid any confusion, we have decided to remove the phrase "informed by Jewell's (2010) guidance" from the manuscript. We believe this will clarify our approach and better align with the inductive nature of the analysis.
